# A Multimodal Closed-Loop Framework for Vital Sign Monitoring and Intelligent Diagnosis of Amusement Ride Passengers Under High-Dynamic Motion

**DOI:** 10.3390/s26134003

**Published:** 2026-06-24

**Authors:** Yikun Wu, Yulong Song, Hao Yang, Ming Zhang

**Affiliations:** 1 School of Design and Art, Beijing Technology and Business University, Beijing 100048, China; 2 School of Computer and Artificial Intelligence, Beijing Technology and Business University, Beijing 100048, China

**Keywords:** motion artifacts, vital-sign quality, physiological-state recognition, closed-loop framework, passenger monitoring, intelligent diagnosis

## Abstract

**Highlights:**

- A two-stage multimodal closed-loop framework was developed for ride passenger monitoring.
- Physiological and kinematic signals were jointly modeled to resist motion artifacts.
- GB 8408-2018 rules enabled interpretable auto-labeling and early warning.
- TAPNet achieved rapid kinematic anomaly screening with high accuracy.
- HS-BANet enabled fine-grained multi-class arrhythmia diagnosis.

**What are the main findings?**

- The heart rate estimation model remained accurate under high dynamics (RMSE 1.18 bpm on HSSH-I; 1.24 bpm on HSSH-II), with high correlation and near-zero Bland–Altman bias.
- TAPNet reached 98.2% test accuracy for kinematic anomaly recognition, and HS-BANet achieved 92.37% accuracy (F1 = 86.87%) for multi-class arrhythmia classification.

**What are the implications of the main findings?**

- A rule-guided + deep learning pipeline can improve reliability and interpretability in non-stationary, motion-corrupted physiological monitoring.
- The framework is suitable for deployable safety early warning and operational support in amusement ride scenarios.

**Abstract:**

High-dynamic amusement ride conditions involving impacts, rapid rotations, and abrupt posture changes introduce severe motion artifacts that degrade vital sign quality and destabilize physiological state recognition. This study aims to develop an engineering-ready closed-loop framework for robust passenger monitoring and intelligent diagnosis. A multimodal sensing and modeling pipeline was designed to jointly leverage physiological signals such as heart rate and SpO_2_ and kinematic measurements, including acceleration, angular rate, velocity, and attitude. Inertial and PPG signals were preprocessed into supervised samples through wavelet multiresolution denoising and coordinate frame unification, while a strapdown inertial navigation system was used to propagate a 12-channel physical quantity sequence. To ensure interpretability and standards compliance, constraints from GB 8408-2018 were translated into executable threshold rules, enabling standards-driven auto-labeling and rule-based early warning. Building on this foundation, three learning modules were developed: a fusion model for high-dynamic heart rate estimation, a CNN–LSTM dynamic-threshold-enhanced network TAPNet for rapid kinematic anomaly screening, and an attention-augmented hybrid model HS-BANet integrating one-dimensional residual blocks, bidirectional LSTM, and multi-head attention for fine-grained arrhythmia classification. Experimental results demonstrated accurate and consistent heart rate estimation with RMSE of 1.18 bpm on HSSH-I and 1.24 bpm on the independent HSSH-II set, strong agreement with training and testing correlations of 0.9928 and 0.9865, and near-zero bias in Bland–Altman analysis. TAPNet achieved 96.9% validation accuracy and 98.2% test accuracy for kinematic anomaly recognition, maintaining robust generalization under class imbalance. HS-BANet enabled multi-class identification of PVC, PAC, VT, SVT, and AF, achieving an accuracy of 92.37%, an F1-score of 86.87%, a precision of 88.45%, a sensitivity of 88.14%, and a specificity of 89.42%. Overall, the proposed two-stage multimodal closed-loop—fast, interpretable early warning based on physical quantity thresholds followed by fine-grained diagnosis from physiological signals—supports stable feature extraction and reliable decision-making under strong motion artifacts and non-stationary dynamics, balancing responsiveness and diagnostic credibility, while showing potential for practical safety early warning and future deployment-oriented operational support in amusement ride scenarios.

## 1. Introduction

With advances in sensing, wireless communications, cloud computing, and embedded intelligence, vital sign monitoring is transitioning from in-hospital, intermittent measurements to multi-scenario, continuous, and intelligent services. Wearable devices and wireless sensor networks can continuously acquire key physiological indicators—such as heart rate, peripheral oxygen saturation (SpO_2_), body temperature, and respiration—and, via Internet of Things (IoT) platforms, enable data aggregation, storage, analysis, and visualization. This technological stack supports early warning for clinical deterioration, remote rehabilitation, and clinical decision support [1,2,3,4,5,6,7]. In parallel, artificial intelligence (AI) methods are further driving health information management beyond “data logging and display” toward risk identification and decision assistance, demonstrating substantial potential for improving clinical efficiency, patient safety, and healthcare quality [4,5,6,7]. Therefore, establishing an end-to-end paradigm of “edge/endpoint sensing—network transmission—cloud/edge intelligent analytics” has become a major technical pathway for remote health monitoring.

Around this paradigm, prior studies have formed a relatively clear research landscape spanning platform architectures and challenges to application forms and algorithmic methods. First, regarding medical IoT architectures and key challenges, Isravel D. P. conducted a systematic review and summarized privacy, security, storage, and transmission challenges in cloud-based medical IoT for clinical data processing and outlined a typical pipeline from sensors to electronic health platforms for vital sign data [8]. Singh B. summarized, from a technology stack perspective, the roles of RFID, Bluetooth, wireless sensor networks (WSN), and ZigBee in remote patient monitoring, emphasizing how cloud-side processing and transmission mechanisms affect system usability and reliability [9]. Second, for the overall profiling of wearable monitoring research, Kristoffersson A. provided a comprehensive analysis of sensor types, sample sizes, acquisition contexts, and methodological choices in wearable sensing studies, noting that many reviews focus on gait/fall detection, while gaps remain regarding “ideal functional form factors” and large-scale datasets [10]. For alarm frameworks, Van Rossum M. C. discussed the effectiveness of threshold-based alarms in monitoring clinical adverse events and argued for adaptive thresholds to enhance early deterioration detection [11]. In system prototyping and engineering pipelines, Misbahuddin S. presented an IoT-based prototype for dynamic vital sign data transmission and highlighted reliability risks of communication links under extreme scenarios [12]. Moreover, monitoring modalities have expanded from wearables to contactless solutions: Mercuri M. proposed radar-based contactless vital sign monitoring and pointed out its application potential in fall detection and gait recognition [13]. Fan Y. emphasized the advantages of wearable devices in convenience and automatic data import and suggested stratified population evaluation to more accurately characterize performance boundaries [14]. In response to public health events, Mohammadzadeh N. and colleagues advanced closed-loop explorations of “real-time monitoring—intelligent analytics—management decision-making” from the perspectives of wearable IoT monitoring, cloud-side analytic services, and epidemic control applications [15]. Meanwhile, Amin S. U. and coauthors systematically discussed deep learning applications, platform-level deployment, and privacy/security issues from the viewpoint of edge intelligence and health IoT/cloud computing [16]. Overall, these studies have established the foundational framework for vital sign monitoring; however, typical applications are largely oriented toward daily life or low-to-moderate intensity conditions, and scenario-specific support for the coupled “vital signs–motion–risk” problem under high-dynamic, strong-disturbance environments remains insufficient.

From an algorithmic and signal-processing perspective, research on preprocessing for inertial and physiological signals provides a methodological reserve for complex scenarios. For acceleration signals, Wu combined wavelets with compressive sensing to improve reconstruction accuracy [17], and Shen proposed an adversarial generative denoising framework to enhance robustness under heavy noise [18]. Hon constructed time-varying filters based on STFT to improve abrupt-change/peak estimation [19], while Zi-you proposed an adaptive boosting wavelet strategy that balances fidelity and efficiency [20]. Under complex vibration and MEMS conditions, Li integrated CEEMDAN with adaptive simulated annealing for parameter optimization [21], Zhang introduced ICA to improve frequency resolution [22], and Jian and Wang reported engineering-relevant conclusions through comparisons among EMD + wavelet preprocessing and filtering schemes [23,24]. For angular rate signals, Qu proposed adaptive denoising using the Haar à trous wavelet to suppress drift while preserving dynamic response [25], and Su combined CEEMDAN with an improved threshold function to extract weak angular rate components [26]. He and Zou improved fidelity from the perspectives of strong-noise detection and suppression of EMD boundary effects, respectively [27,28]. Huang and Liu further focused on the diffusion of integration errors in angular rate signals and proposed a fusion estimation and denoising strategy incorporating jerk constraints [29]. For high-precision gyroscope scenarios, Sun et al. significantly reduced random-walk noise using hybrid Kalman filtering and adaptive moving-average schemes [30], and the nonlinear filtering models of Bodile and Rao provided a transferable state-estimation perspective [31]. For physiological signals, Geng combined EEMD with NLMS to remove motion artifacts in PPG [32]; Cheng and Yu integrated CEEMDAN with entropy features to improve dynamic heart rate estimation [33]; Rankawat proposed an SQI-based fusion stabilization strategy [34]; and Pandey and Chao suppressed drift from the perspective of hardware front-end compensation [35]. For ECG and SpO_2_/PPG processing, Zhao and Xu, Sasirekha, Tejaswi, Durrani, as well as Zhan, Rao, Tang, Xu, and others, proposed methods, including CEEMDAN-based component selection, wavelet + digital filtering, RLS simulation, multi-stage adaptive filtering, VMD + ICA separation, and neural-network-based recovery, providing support for high-fidelity monitoring under complex noise conditions [36,37,38,39,40,41,42,43].

In contrast to studies primarily targeting “medical/daily-life scenarios,” amusement rides are characterized by high speed, high acceleration, strong vibration, and pronounced attitude variations, leading to more prominent structural and public safety risks. Iino and Nakao pointed out that fatigue cracks in roller-coaster main shafts can be difficult to detect with conventional inspection approaches and may cause fracture accidents, highlighting the importance of periodic inspection for critical components [44]. Related failure analyses also emphasize the compounded risk of fatigue damage induced by welding defects and residual stresses [45]. In active safety and passenger protection, Zampella proposed restraint systems integrating contact sensors to enhance active safety [46], and Shad and Hasan, through accident analyses, emphasized stress concentration–prone weak regions as key factors in structural design and suggested optimizing limit/constraint structures [47]. However, existing amusement ride safety research remains largely centered on the equipment itself and rarely incorporates passenger vital signs as potential early warning cues into a unified monitoring and diagnostic framework. Moreover, in the limited body of work involving physiological measurements, studies often remain at the level of basic recording or experience evaluation; they typically lack systematic preprocessing, feature modeling, and abnormality identification mechanisms designed specifically for high-dynamic conditions—thereby limiting engineering-grade early warning and intervention.

Motivated by these gaps, this study targets high-dynamic operating scenarios in amusement rides and investigates multimodal vital sign measurement, high-dynamic signal preprocessing, intelligent diagnosis, and risk early warning. The main contributions are summarized as follows:

Motivated by these gaps, this study targets high-dynamic operating scenarios in amusement rides and investigates multimodal vital sign measurement, high-dynamic signal preprocessing, intelligent diagnosis, and risk early warning. The main contribution of this work lies not in proposing entirely new standalone algorithmic modules but in developing a scenario-oriented methodological framework for high-dynamic amusement ride passenger monitoring under severe motion artifacts and non-stationary disturbances. Specifically, this study makes three contributions. First, it establishes a standards-guided two-stage closed-loop framework that links rapid kinematic risk screening with fine-grained physiological diagnosis. Second, it translates GB 8408-2018 [48] safety constraints into executable threshold rules for interpretable auto-labeling and early warning, rather than using them only as post hoc engineering references. Third, it formulates a multimodal sensing and modeling pipeline tailored to strong motion artifacts and non-stationary dynamics, enabling coordinated use of inertial and physiological signals in high-dynamic environments. In this study, the target problem is formulated as a two-stage task. The first stage performs rapid abnormal state screening based on kinematic physical quantities for early warning, whereas the second stage performs fine-grained physiological diagnosis on flagged segments using physiological signals.

The remainder of this paper is organized as follows. Section 2 introduces inertial and PPG signal processing methods, including inertial data preprocessing and denoising, as well as PPG denoising and heart rate estimation. Section 3 presents the proposed recognition and diagnostic methods based on inertial and physiological signals in high-dynamic environments. Section 4 reports the experimental results and discussion, including model evaluation and comparative analysis. Finally, Section 5 concludes the paper and discusses future research directions.

## 2. Materials and Methods

This chapter focuses on inertial-sensor signal processing for amusement ride passengers under high-dynamic motion. The goal is to improve the quality and stability of acceleration and angular rate measurements via error modeling, Allan variance analysis, wavelet denoising, and attitude estimation. We first introduce the motion–heart rate dataset collected in this work as the basis for validation. We then establish tri-axial error models and conduct Allan variance analysis to identify multiple inertial noise types at the source. For data preprocessing, coordinate alignment, gravity removal, and wavelet multiresolution analysis are adopted to effectively suppress noise, providing cleaner inputs for learning-based models. Finally, a strapdown inertial navigation system (SINS) is used to estimate velocity, attitude, and position with high accuracy, producing the physical quantity time series required for subsequent anomaly detection.

**Figure 1:**
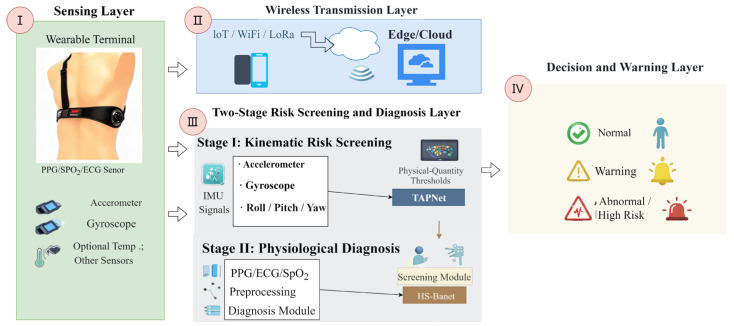
Architecture of the wearable passenger physiological signal monitoring and warning system for amusement rides. I: sensing layer; II: wireless transmission layer; III: two-stage risk screening and diagnosis layer; IV: decision and warning layer. The arrows indicate the signal transmission, data processing, and decision-making flow from sensor acquisition to wireless communication, risk diagnosis, and warning output.

Figure 1 presents the high-level architecture of the proposed multimodal closed-loop passenger monitoring framework. The system consists of four parts: the sensing layer, the wireless transmission and edge/cloud integration layer, the two-stage risk screening and diagnosis layer, and the decision and warning layer. Multimodal physiological and inertial signals are first acquired by the wearable terminal and then transmitted through the wireless communication pathway to the upper-level platform. After preprocessing, the uploaded data are processed through Stage I kinematic risk screening and Stage II physiological diagnosis, and the final outputs are mapped to different warning levels for passenger state monitoring in high-dynamic amusement ride scenarios.

### 2.1. Inertial Data Preprocessing and Denoising

A fundamental challenge in inertial navigation is that errors accumulate and propagate through integration. Therefore, before attitude and trajectory reconstruction, inertial data must be organized consistently, its noise characteristics must be identified, and error terms must be parameterized to enable feasible preprocessing and compensation strategies. This section first describes the data sources and acquisition settings, specifying the signal types, sampling rates, and usage protocols for subsequent analyses. Then, based on error models and Allan variance analysis, the principal stochastic error terms of the accelerometer and gyroscope are characterized, and their parameters are identified. Finally, after denoising and error characterization, SINS is employed to recursively estimate attitude, velocity, and position, providing the physical variables needed for later state determination and safety-threshold analysis.

#### 2.1.1. Datasets

This study aims to validate the effectiveness and generalizability of the proposed heart rate estimation model in high-dynamic scenarios. Two self-collected datasets are constructed and used: HSSH-I and HSSH-II. Specifically, HSSH-I is used for model training and sample scale augmentation, while HSSH-II is reserved for independent validation to ensure that the evaluation reflects true generalization under unseen subject/unseen condition settings. Both datasets are obtained from synchronized measurements collected during subjects’ rides on high-speed amusement facilities. They include physiological information characterizing the subject state (e.g., heart rate and respiration) and kinematic observation channels representing motion disturbances (e.g., tri-axial acceleration).

**Figure 2:**
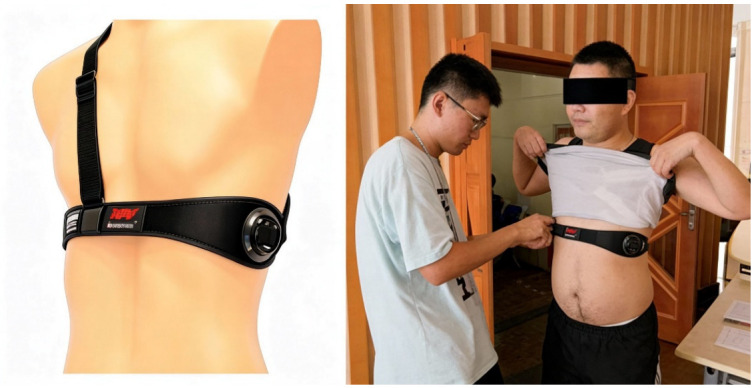
Schematic of the physiological recorder and on-site wearing procedure.

Data acquisition is performed using the BioHarness portable physiological measurement system (Zephyr Technology Corporation, Annapolis, MD, USA; supplied by BIOPAC Systems, Inc., Goleta, CA, USA) was used to collect physiological signals, which synchronously telemeters and logs heart rate, respiration rate, acceleration, ECG, and other signals, and exports them to a computer for storage. The sampling rates are configured as follows: 1 Hz for heart rate/respiration and 18 Hz for acceleration. To ensure stable measurements under high-dynamic conditions, subjects complete chest-strap placement, tightening, and positional calibration before acquisition. The wearing position and fixation procedure are illustrated in Figure 2. This chest-strap configuration maintains reliable contact and attachment under severe motion and impacts, thereby reducing signal-quality fluctuations caused by loosening.

In terms of data usage, HSSH-I serves as the training set to learn heart rate dynamics under motion interference and to enhance the model’s adaptability to complex artifacts, while HSSH-II is used as an independent test set to evaluate robustness and generalization across different subjects and ride sessions. To ensure comparability between training and validation, the same windowing and evaluation pipeline is applied to both datasets: signals are segmented using a fixed-length sliding window, and supervision labels and model input features are generated for each window, forming a consistent end-to-end “training–validation” experimental loop.

To further clarify the composition and acquisition conditions of the two datasets, both HSSH-I and HSSH-II were collected using a standardized 12 min continuous monitoring protocol under real outdoor amusement ride conditions. HSSH-I contains 24 experimental samples and includes 10 male and 14 female participants. Its acquisition protocol consists of 1 min of resting state, 2 min of initial motion adaptation on a slide, 3 min of moderate-intensity circular stimulation on a carousel, 2 min of slow walking for recovery, 3 min of high-intensity sudden acceleration under roller-coaster-like conditions, and 1 min of cooling-down experience on a sightseeing vehicle. In contrast, HSSH-II contains 23 experimental samples, including 17 male and 6 female participants. Its protocol consists of 1 min of resting state, 2 min of warm-up on a slowly rotating ride, 4 min of sustained moderate-intensity stimulation on a carousel, 4 min of low-speed motion on a sightseeing mini-train, and 1 min of resting recovery. To simulate seat-supported passenger conditions, participants in HSSH-II were additionally instructed to hold the safety handle during the final stage of the ride process. Compared with HSSH-I, HSSH-II therefore represents a different motion-exposure pattern with relatively more sustained but lower-intensity dynamic stimulation, providing an additional basis for evaluating model robustness and cross-condition generalization.

#### 2.1.2. Noise Characterization

In high-dynamic amusement ride scenarios, impacts, vibration, and rapid posture changes amplify high-frequency noise and bias fluctuation in inertial measurements, which directly degrades attitude propagation and integration-based estimates. Therefore, before navigation state estimation, inertial observations were first characterized using standard tri-axial gyroscope and accelerometer error models, and Allan variance analysis was applied to identify the dominant stochastic error components across different time scales. Since these formulations are standard in inertial navigation literature, only the key equations and their role in the present framework are retained here.

(1) $$\left[\begin{array}{c} \omega_{m_{x}}\\ \omega_{m_{y}}\\ \omega_{m_{z}} \end{array}\right]=\left[\begin{array}{ccc} S_{b}x & M_{xy} & M_{xz}\\ M_{yx} & S_{b}y & M_{yz}\\ M_{zx} & M_{zy} & S_{b}z \end{array}\right]\left[\begin{array}{c} \omega_{t_{x}}\\ \omega_{t_{y}}\\ \omega_{t_{z}} \end{array}\right]+\left[\begin{array}{ccc} G_{xx} & G_{xy} & G_{xz}\\ G_{yx} & G_{yy} & G_{yz}\\ G_{zx} & G_{zy} & G_{zz} \end{array}\right]\left[\begin{array}{c} a_{x}\\ a_{y}\\ a_{z} \end{array}\right]+\left[\begin{array}{c} G_{x}^{2}a_{x}a_{z}\\ G_{y}^{2}a_{z}a_{y}\\ G_{z}^{2}a_{z}a_{y} \end{array}\right]+\left[\begin{array}{c} b_{x}\\ b_{y}\\ b_{z} \end{array}\right]+\left[\begin{array}{c} Tb_{x}\\ Tb_{y}\\ Tb_{z} \end{array}\right]+\left[\begin{array}{c} \eta_{x}\\ \eta_{y}\\ \eta_{z} \end{array}\right]$$

In Equation (1), $\omega_{m}$ denotes the measured angular rate vector on each axis, $\omega_{t}$ denotes the true angular rate vector, and a denotes the acceleration vector. S represents scale factor errors, M represents mounting/misalignment errors, and G represents g sensitivity-related drift (including cross-axis g-sensitivity terms). b is the bias term, Tb represents temperature-induced drift, and η is the random noise term.

(1) Tri-axial accelerometer error model

The tri-axis accelerometer error model is given by Equation (2):

(2) $$\left(\begin{array}{c} a_{mx}\\ a_{my}\\ a_{mz} \end{array}\right)=\left(\begin{array}{ccc} S_{x} & M_{xy} & M_{xz}\\ M_{yx} & S_{y} & M_{yz}\\ M_{zx} & M_{zy} & S_{z} \end{array}\right)\left(\begin{array}{c} a_{tx}\\ a_{ty}\\ a_{tz} \end{array}\right)+\left(\begin{array}{c} b_{x}\\ b_{y}\\ b_{z} \end{array}\right)+\left(\begin{array}{c} Ta_{x}\\ Ta_{y}\\ Ta_{z} \end{array}\right)+\left(\begin{array}{c} A_{CGx}\\ A_{CGy}\\ A_{CGz} \end{array}\right)+\left(\begin{array}{c} \eta_{x}\\ \eta_{y}\\ \eta_{z} \end{array}\right)$$

In Equation (2), $a_{m}$ and $a_{t}$ are the measured and true acceleration vectors, respectively; S and M denote scale factor and misalignment errors; b is the bias; Ta is the temperature-induced drift; and η is the random noise term.

**Figure 3:**
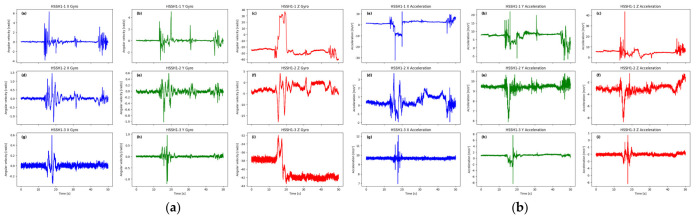
Synchronous raw data from the gyroscope and accelerometer. (**a**) Raw angular rate signals from the gyroscope and (**b**) tri-axial acceleration signals from the accelerometer. The blue, green, and red curves represent the X-, Y-, and Z-axis signals, respectively.

Based on the measured angular rate and acceleration sequences, the above error models were implemented in MATLAB 2022 to evaluate the influence of sensor imperfections on inertial observations. The corresponding raw signals are shown in Figure 3. As illustrated, scale factor drift, cross-axis misalignment, bias fluctuation, and temperature disturbance can cause both instantaneous deviation and cumulative integration drift, which motivates the need for further time-scale noise characterization.

To quantify the stochastic behavior of inertial measurements across different averaging times, Allan variance analysis was employed. In the present study, Allan variance is used mainly to distinguish dominant short-term and long-term noise processes rather than to provide a full theoretical derivation of inertial-noise taxonomy.

**Figure 4:**
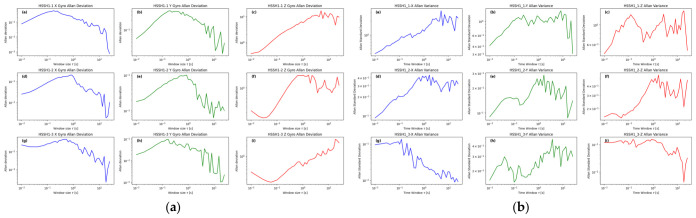
Allan deviation and Allan variance plots for the gyroscope and accelerometer. (**a**) Allan deviation curves of angular velocity measured by the gyroscope and (**b**) Allan variance curves of acceleration measured by the accelerometer. In all subplots, the blue, green, and red curves denote the X-, Y-, and Z-axis signals, respectively.

The Allan deviation curves obtained for the gyroscope and accelerometer are shown in Figure 4. The results indicate that short-term behavior is primarily dominated by high-frequency fluctuation and random-walk-like noise, whereas with increasing averaging time the contribution of bias instability and low-frequency drift becomes more apparent. These observations provide direct guidance for the subsequent denoising and signal-conditioning procedure.

After error characterization, inertial data preprocessing was performed before deep learning modeling. Specifically, coordinate unification and mirror transformation were applied to eliminate inconsistency caused by different wearing orientations; gravity was estimated and removed to obtain net motion acceleration; and discrete wavelet transform (DWT)-based denoising was used to suppress the remaining high-frequency noise and transient impacts. In this work, a Daubechies wavelet with a maximum decomposition level of 3 was adopted for wavelet multiresolution analysis, which was found to provide a good balance between noise suppression and structural preservation for non-stationary inertial signals.

**Figure 5:**
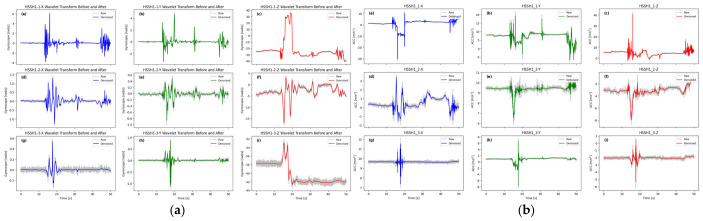
Wavelet-denoised tri-axial signals. (**a**) Angular rate; (**b**) acceleration.

The denoising results are shown in Figure 5. Compared with the raw measurements, the processed signals exhibit reduced high-frequency disturbance while preserving the major motion trend and informative low-frequency structure, making them more suitable as inputs for subsequent navigation state estimation and learning-based recognition.

In summary, inertial error characterization and multiresolution denoising provided cleaner and more stable motion observations for subsequent SINS computation. The processed angular rate and specific force signals were then used to estimate attitude, velocity, and position, producing the physical quantity time series required for later threshold recognition and state diagnosis.

#### 2.1.3. Strapdown Inertial Navigation System

**Figure 6:**
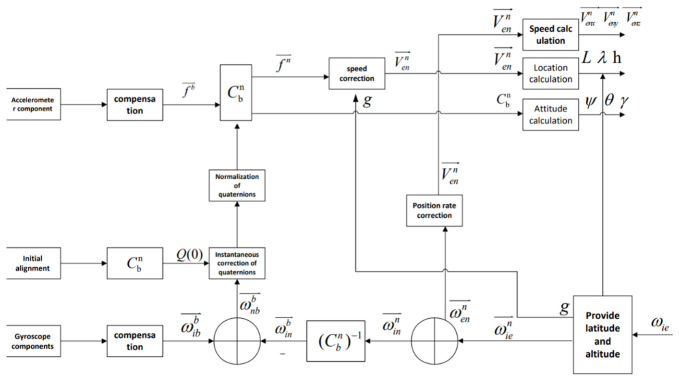
SINS computation flow.

To obtain velocity, attitude, and position from tri-axial angular rate and acceleration measurements, a strapdown inertial navigation system (SINS) was employed. Since quaternion-based SINS updating is a standard inertial navigation procedure, this section focuses only on the computation flow and output variables directly used in subsequent recognition tasks. The overall processing flow is shown in Figure 6.

During initialization, the initial attitude matrix was established through alignment using gravity and Earth-rate information in the navigation frame.

(1) Initial alignment

During initial alignment, the objective is to establish the initial transformation matrix between the body frame b and the navigation frame n, denoted by $C_{b}^{n}$. Given known latitude L and longitude λ, the gravity vector $g^{n}$ and Earth rotation rate $\omega_{ie}^{n}$ in the navigation frame are computed as Equations (3) and (4):

(3) $${\vec{g}}^{n}=\left[\begin{array}{c} g_{x}^{n}\\ g_{y}^{n}\\ g_{z}^{n} \end{array}\right]=\left[\begin{array}{c} 0\\ 0\\ -g \end{array}\right]$$

(4) $${\vec{\omega }}_{ie}^{n}=\left[\begin{array}{c} \omega_{iex}^{n}\\ \omega_{iey}^{n}\\ \omega_{iez}^{n} \end{array}\right]=\left[\begin{array}{c} 0\\ \omega_{ie}\cos L\\ \omega_{ie}\sin L \end{array}\right]$$

A vector $\gamma^{n}$ is constructed via the cross product in Equation (5):

(5) $$\vec{\gamma }={\vec{g}}^{n}\times {\vec{\omega }}_{ie}^{n}$$

The relationship between the navigation frame n and the body frame b is expressed by $C_{b}^{n}$. Using the measured vectors $g^{b}$ and $\omega_{ie}^{b}$ from sensors together with $g^{n}$, $\omega_{ie}^{n}$, and $\gamma^{n}$, the initial DCM can be constructed by Equation (6):

(6) $$C_{b}^{n}={\left[\begin{array}{c} {\left({\vec{g}}^{n}\right)}^{t}\\ {\left({\vec{\omega }}_{ie}^{n}\right)}^{t}\\ {\left({\vec{\gamma }}^{n}\right)}^{t} \end{array}\right]}^{-1}\left[\begin{array}{c} {\left({\vec{g}}^{b}\right)}^{t}\\ {\left({\vec{\omega }}_{ie}^{b}\right)}^{t}\\ {\left({\vec{\gamma }}^{b}\right)}^{t} \end{array}\right]$$

Here, $g^{n}$ is computed by Equation (13) and $\omega_{ie}^{n}$ by Equation (14), while $g^{b}$ and $\omega_{ie}^{b}$ are obtained from sensor outputs. After solving Equation (16), initial alignment of the attitude matrix is completed.

By substituting the parameters from Equations (4) and (5) into Equation (6), the expression for $C_{b}^{\;n}$ can be derived; the resulting form is given in Equation (7).

(7) $$\left.\begin{array}{l} T_{11}=\frac{\sec L}{g\omega_{ie}}(\omega_{iez}^{b}g_{y}^{b}-\omega_{iey}^{b}g_{z}^{b}),T_{12}=\frac{\sec L}{g\omega ie}(\omega_{iex}^{b}g_{z}^{b}-\omega_{iez}^{b}g_{x}^{b}),T_{13}=\frac{\sec L}{g\omega ie}(\omega_{iey}^{b}g_{x}^{b}-\omega_{iex}^{b}g_{y}^{b})\\ T_{21}=\frac{g_{x}^{b}}{g}\tan L+\frac{\omega_{iex}^{b}}{\omega_{ie}}\sec L,\;\;\;\;T_{22}=\frac{g_{y}^{b}}{g}\tan L+\frac{\omega_{iey}^{b}}{\omega_{ie}}\sec L,\;\;\;\;\;\;T_{23}=\frac{g_{z}^{b}}{g}\tan L+\frac{\omega_{iez}^{b}}{\omega_{ie}}\sec L\\ T_{31}=-\frac{g_{x}^{b}}{g},\;\;\;\;\;\;\;\;\;\;\;\;\;\;\;\;\;\;\;\;\;\;\;\;\;\;\;\;T_{32}=-\frac{g_{y}^{b}}{g},\;\;\;\;\;\;\;\;\;\;\;\;\;\;\;\;\;\;\;\;\;\;\;\;\;\;\;\;\;\;\;\;T_{33}=-\frac{g_{z}^{b}}{g} \end{array}\right\}$$

Here, $g_{x}^{b}$, $g_{y}^{b}$, and $g_{z}^{b}$ can be approximately replaced by the accelerometer outputs $f_{x}^{b}$, $f_{y}^{b}$, and $f_{z}^{b}$. Once Equation (17) is solved, the initial alignment of the attitude matrix is completed.

(2) Instant correction of quaternion Q

Three common approaches are used for attitude updating: Euler-angle methods, direction cosine matrix methods, and quaternion methods. Euler-angle methods are easy to interpret and implement but suffer from singularities and cumulative error risks. DCM methods have better numerical stability but still involve cumulative errors and higher computational complexity. Compared with the above methods, quaternion-based updating provides improved numerical stability and better suppression of cumulative errors; therefore, we adopt quaternion updating.

A quaternion consists of a real unit and three imaginary units and is expressed by Equation (8):

(8) $$Q=q_{0}+q_{1}\vec{i}+q_{2}\vec{j}+q_{3}\vec{k}$$

Quaternion updating can be implemented by solving the quaternion differential equation in Equation (9):

(9) $$\left[\begin{array}{c} q_{0}^{\prime }\\ q_{1}^{\prime }\\ q_{2}^{\prime }\\ q_{3}^{\prime } \end{array}\right]=\frac{1}{2}\left[\begin{array}{cccc} 0 & -\omega_{nbx}^{b} & -\omega_{nby}^{b} & -\omega_{nbz}^{b}\\ \omega_{nbx}^{b} & 0 & \omega_{nbz}^{b} & \omega_{nby}^{b}\\ \omega_{nby}^{b} & -\omega_{nbz}^{b} & 0 & \omega_{nbx}^{b}\\ \omega_{nbz}^{b} & \omega_{nby}^{b} & -\omega_{nbx}^{b} & 0 \end{array}\right]\left[\begin{array}{c} q_{0}\\ q_{1}\\ q_{2}\\ q_{3} \end{array}\right]$$

where $q=[q_{0},q_{1},q_{2},q_{3}{]}^{⊤}$, and Ω(ω) is the standard quaternion kinematic matrix constructed from the measured angular rates. The initial condition Q(0) is required and is obtained from the elements of the initial attitude matrix $C_{b}^{n}(0)$.

(3) Instant correction of speed

Velocity updating includes two steps.

First, the specific force coordinate transformation is performed. Using the accelerometer output $f^{b}$ and $C_{b}^{n}$, the specific force in the navigation frame is computed by Equation (10):

(10) $$\vec{f^{n}}=C_{b}^{n}\vec{f^{b}}$$

Second, the velocity differential equation is solved. Using Earth rotation and transport rates along with $f^{n}$, the velocity dynamics can be written in the generic form of Equation (11):

(11) $$\left[\begin{array}{c} {\dot{V}}_{x}^{p}\\ {\dot{V}}_{y}^{p}\\ {\dot{V}}_{z}^{p} \end{array}\right]=\left[\begin{array}{ccc} 0 & 2\omega_{iez}^{p}+\omega_{ep:}^{p} & -\left(2\omega_{iey}^{p}+\omega_{epy}^{p}\right)\\ -\left(2\omega_{ie:}^{p}+\omega_{ep:}^{p}\right) & 0 & 2\omega_{iex}^{p}+\omega_{epx}^{p}\\ 2\omega_{iey}^{p}+\omega_{epy}^{p} & -\left(2\omega_{iex}^{p}+\omega_{epx}^{p}\right) & 0 \end{array}\right]\left[\begin{array}{c} V_{x}^{p}\\ V_{y}^{p}\\ V_{i}^{p} \end{array}\right]$$

where $V^{n}$ is the velocity in the navigation frame and $\omega_{en}^{n}$ is the transport rate.

(4) Attitude-angle computation

The attitude angles can be computed from the attitude matrix elements using Equation (12):

(12) $$\;\left.\begin{array}{c} \psi =\arctan\frac{T_{12}}{T_{22}}\\ \;\;\;\;\theta =\arcsin T_{32},\;\\ \gamma =\arctan(-\frac{T_{31}}{T_{33}}) \end{array}\right\}$$

(5) Position update

Longitude, latitude, and height can be computed from velocities using Equation (13):

(13) $$L=\frac{V_{eny}^{n}}{R_{yn}+h},\;\lambda =\frac{V_{enx}^{n}}{\left(R_{xn}+h\right)\cos L},\;h=V_{enz}^{n}$$

where R is the Earth radius parameter and $V_{en},V_{nn},V_{un}$ are the east, north, and up velocity components.

The above procedures provide accurate data support for subsequent roller-coaster trajectory fitting and visualization, and they also establish the basis for visualizing attitude at specific trajectory points, displaying acceleration-based safety judgments, and generating overall operational visualizations.

In the present framework, the main purpose of SINS is not to provide a full navigation theory derivation but to generate unified frame physical quantities for subsequent threshold recognition and multimodal diagnosis. Specifically, the SINS outputs include tri-axial velocity, attitude angles, transformed acceleration, and related motion variables, which are further organized into the 12-channel physical quantity sequence used in later kinematic anomaly recognition and physiological-signal-assisted diagnosis.

**Figure 7:**
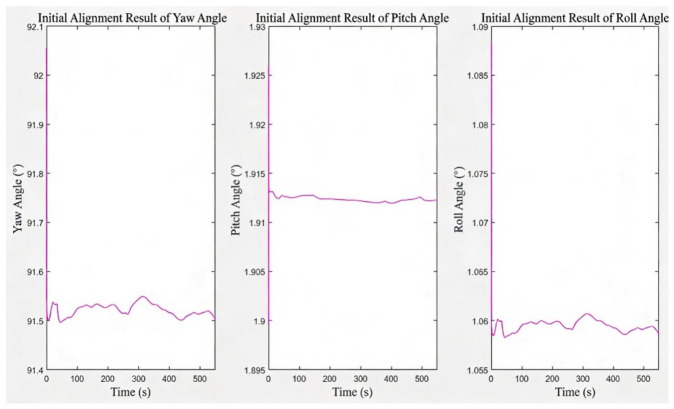
Analysis of initial alignment results.

**Figure 8:**
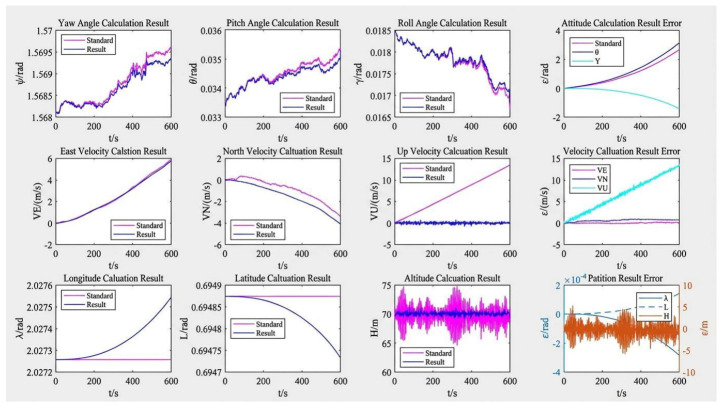
Outputs after attitude estimation.

The implemented SINS solution showed stable alignment and reasonable motion-state outputs, as illustrated in Figure 7 and Figure 8. Figure 7 presents the initial alignment result, where the attitude angles fluctuate slightly around stable values, indicating effective initialization. Figure 8 further shows the outputs of attitude information, spatial coordinates, and tri-axial velocities. These results confirm that the estimated motion states are sufficiently stable for subsequent feature construction, threshold analysis, and multimodal fusion.

In summary, after inertial error characterization and wavelet denoising, this work employs SINS to recursively estimate attitude, velocity, and position, producing unified frame physical quantity sequences such as attitude angles, tri-axial angular rate, transformed acceleration, and motion velocity. These outputs serve as direct inputs for later threshold-based recognition and state diagnosis and also provide key motion priors for physiological signal processing. In high-dynamic amusement scenarios, where heart rate observation is strongly affected by motion artifacts and contact-condition variation, inertial measurements help quantify disturbance intensity and temporal motion patterns, thereby supporting multimodal alignment, artifact suppression, and feature fusion in the following heart rate analysis stage.

### 2.2. Heart Rate Signal Preprocessing and Denoising

In high-dynamic amusement scenarios, PPG/heart rate signals are strongly affected by body vibration, changes in skin contact pressure, and motion artifacts, exhibiting pronounced non-stationary noise. To improve the robustness of heart rate estimation, we incorporate the net motion observations and attitude estimates obtained in Section 2.1 as motion priors. Specifically, inertial outputs are used to characterize disturbance intensity and direction changes, enabling cross-modal time alignment and unified resampling. During feature construction, the frequency-domain representation of PPG is fused with the disturbance characterization from acceleration/angular rate, thereby suppressing the influence of motion artifacts on spectral-peak localization and heart rate tracking. This section presents a complete pipeline centered on time alignment—multi-stage preprocessing—time–frequency feature construction, consistent with the input format of subsequent models.

#### 2.2.1. Multi-Stage Preprocessing and Feature Construction

(1) Data sources and sliding window definition

Two self-collected datasets, HSSH-I and HSSH-II, are used. HSSH-I is used for training and sample augmentation, and HSSH-II is used as an independent test set for generalization evaluation. Data are synchronously recorded via the BIOPAC BioHarness portable physiological measurement system, covering heart rate, respiration, acceleration, ECG, and other signals. The heart rate and respiration-rate sampling frequency is 1 Hz, and the acceleration sampling frequency is 18 Hz (used to characterize motion intensity and disturbance rhythm).

To unify feature construction and model inputs, fixed sliding windows are used for sample segmentation. Let the ground truth and estimated heart rates of the i-th window be HR(i) and $\hat{HR}(i)$, respectively, for i=1,2,…,I. A window length of 8 s and a step size of 2 s (i.e., 6 s overlap) are adopted to balance time–frequency resolution and sample size.

(2) Time alignment and unified sampling rate

Because the raw sampling rates of multimodal signals are inconsistent, and PPG/ECG may have higher native rates or different output timing, directly applying STFT would lead to inconsistent frequency grids and incomparable feature dimensions. Therefore, cross-modal alignment is performed using timestamps, and signals used for feature construction (PPG and tri-axial acceleration) are resampled to a unified sampling rate $f_{s}=25\;Hz$. Acceleration (18 Hz) is resampled via interpolation with anti-aliasing constraints, and PPG is resampled to the same $f_{s}$ after denoising to ensure that each 8 s window corresponds to 200 samples. The number of discrete points in each 8 s window is thus

(14) $$N_{w}=f_{s}\times 8=25\times 8=200$$

Equation (14) ensures that the subsequent “8 s window → 200-point sequence” mapping is consistent across modalities.

(3) Heart rate band preservation and normalization

Within each window, the PPG signal is denoised via wavelet denoising and/or bandpass constraints, focusing on the dominant heart rate band of 0.4–4 Hz to suppress low-frequency baseline drift and high-frequency environmental noise. Then, Z-score normalization is applied to each PPG channel to enforce zero mean and unit variance, reducing adverse effects from inter-subject and wearing-condition variability.

Let the n-th PPG channel in window i be $S_{n}^{\left(i\right)}(t)$ for n=1,…,N, and the tri-axial acceleration be $A_{m}^{\left(i\right)}(t)$ for m∈{x,y,z}. After normalization, multi-channel PPG is fused along the channel dimension (e.g., by averaging) to form a single sequence:

(15) $$S^{\left(i\right)}(t)=\frac{1}{N}\sum_{n=1}^{N}S_{n}^{\left(i\right)}(t).$$

Equation (15) defines a unified PPG representation for subsequent time–frequency analysis.

(4) STFT and power spectral density (PSD) features

To capture time-varying heart rate characteristics under high dynamics, short-time Fourier transform (STFT) is employed to construct time–frequency representations. For each 8 s window, the time series length is $N_{w}=200$. A Hann window is applied to reduce spectral leakage. To obtain a finer frequency grid, the FFT length is set to $N_{FFT}=2048$ via zero-padding, yielding the frequency resolution.

(16) $$\Delta f=\frac{f_{s}}{N_{FFT}}=\frac{25}{2048}\approx 0.0122\;\mathrm{Hz}\approx 0.73\;\mathrm{bpm}.$$

Equation (16) clarifies the origin of the frequency-bin resolution used in subsequent spectral features.

Let the PSD of PPG in window i be $P_{S}^{\left(i\right)}(f)$, and the PSD of acceleration on axis m be $P_{A_{m}}^{\left(i\right)}(f)$. To improve robustness to random motion directions, the tri-axial acceleration PSDs are averaged:

(17) $$P_{A}^{\left(i\right)}(f)=\frac{1}{3}\sum_{m\in \{x,y,z\}}P_{A_{m}}^{\left(i\right)}(f).$$

Equation (17) defines a direction-robust motion-spectrum representation.

The PSDs are further normalized in magnitude (e.g., min–max or energy normalization) to stabilize input scales, and only the heart-rate-relevant frequency range of 0.6–3.3 Hz (corresponding to 36–198 bpm) is retained. Given Δf≈0.0122 Hz in Equation (26), the number of frequency bins in this band is approximately

(18) $$C\approx \left\lfloor \frac{3.3-0.6}{0.0122}\right\rfloor +1\approx 222$$

Equation (18) explains the source of the “222-dimensional” frequency grid used as model input.

Consequently, each window yields a 1×C PPG spectral feature $P_{S}^{\left(i\right)}\in R^{1\times 222}$ and a 1×C acceleration spectral feature $P_{A}^{\left(i\right)}\in R^{1\times 222}$.

(5) Motion intensity indicator

In addition to frequency-domain PSD features, a motion intensity scalar is introduced to characterize overall body-motion strength. For each acceleration axis, the Hilbert envelope $e_{m}^{\left(i\right)}(t)$ is computed, and the window-averaged intensity is defined as:

(19) $$I_{A}(i)=\frac{1}{3}\sum_{m\in \{x,y,z\}}\frac{1}{T}\int_{t\in \mathrm{window}.i}e_{m}^{\left(i\right)}(t)dt,T=8s.$$

Equation (19) provides a compact intensity descriptor complementary to spectral features.

(6) Summary of final input features

For each window i, the model input comprises three components: the PPG spectral feature $P_{S}^{\left(i\right)}\in R^{1\times 222}$, the acceleration spectral feature $P_{A}^{\left(i\right)}\in R^{1\times 222}$, and the motion intensity scalar $I_{A}(i)$. All samples follow the same windowing strategy (8 s length, 2 s step), ensuring consistent preprocessing and comparability between HSSH-I and HSSH-II.

#### 2.2.2. Model Architecture and Loss Function Design

(1) Model architecture

After feature construction, $P_{S}^{\left(i\right)}$ and $P_{A}^{\left(i\right)}$ are stacked along the modality dimension to form a 2D input tensor:

(20) $$X^{\left(i\right)}=\left[\begin{array}{c} P_{S}^{\left(i\right)}\\ P_{A}^{\left(i\right)} \end{array}\right]\in {\mathbb{R}}^{2\times 222}.\;$$

Equation (20) defines the network input in the “modality × frequency” space.

In the heart rate estimation branch, PPG and acceleration were used as the direct model inputs because this module was designed to focus on motion artifact suppression in PPG under high-dynamic conditions. In our implementation, acceleration was selected as the primary motion reference signal for this branch, since it directly reflects vibration intensity and contact-related disturbance that are closely coupled with PPG corruption.

**Figure 9:**
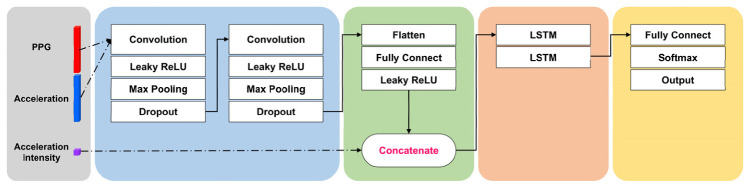
Model architecture. The red, blue, and purple input branches represent the PPG signal, acceleration signal, and acceleration intensity, respectively. The light-blue, green, orange, and yellow modules denote the convolutional feature extraction module, feature fusion module, LSTM temporal modeling module, and fully connected output module, respectively. The arrows indicate the data-flow direction, and the dashed/dash-dotted arrows indicate the input and feature-fusion paths of different signal branches.

It should be noted, however, that gyroscope-derived motion information was not excluded from the overall framework. Instead, tri-axial angular velocity and attitude-related variables were incorporated into the physical quantity-based abnormal state screening branch, where a richer multi-channel kinematic representation was used for rapid risk screening and threshold-based recognition. Therefore, the use of PPG and acceleration in Figure 9 reflects the task-specific input design of the heart rate estimation module, rather than the omission of gyroscope information from the full system.

As illustrated in Figure 9, a 2D CNN is first applied to extract cross-modal coupling patterns on the modality–frequency plane (e.g., co-variations between motion artifacts and heart rate spectral peaks). Then, a 1D CNN along the frequency axis further refines peak morphology and local bandwidth features. Next, a short sequence composed of multiple adjacent windows is fed into a two-layer LSTM (with sequence length set to 6) to model the temporal evolution of heart rate. Finally, a fully connected layer outputs a C=222-dimensional logit vector, and a Softmax layer produces the predicted probability distribution over frequency bins:

(21) $${\hat{y}}^{\left(i\right)}=\mathrm{softmax}(z^{\left(i\right)})\in {\mathbb{R}}^{222}.\;$$

In Equation (21), the c-th element ${\hat{y}}_{c}^{\left(i\right)}$ represents the probability that the heart rate in window i falls into the c-th frequency bin.

To mitigate overfitting, dropout is introduced in the CNN and LSTM modules (with rates specified by experimental settings), while maintaining identical preprocessing and input organization for both HSSH-I and HSSH-II.

(2) Distributional learning objective based on KL divergence

Heart rate estimation is formulated as a distribution prediction problem over the frequency grid. Let the target distribution be $y^{\left(i\right)}\in R^{222}$ and the predicted distribution be ${\hat{y}}^{\left(i\right)}$. The KL divergence loss is defined as

(22) $$\xi (i)=D_{KL}[\Gamma_{i}(y^{\left(i\right)}\|{\hat{y}}^{\left(i\right)})]=\sum_{c=1}^{C}y_{c}^{\left(i\right)}\log\frac{y_{c}^{\left(i\right)}}{{\hat{y}}_{c}^{\left(i\right)}}.$$

Equation (22) allows the ground truth to be expressed as a distribution rather than a one-hot label, which better matches practical conditions under high dynamics where the PPG spectral peak may broaden and its location may slightly drift.

(3) Gaussian-weighted KL

In high-dynamic environments, even with ECG reference heart rate, slight “frequency-bin mismatch” can arise due to pulse transit delay and motion artifacts causing peak shifts, quantization effects from the finite frequency grid, and short-window estimation under non-stationary disturbances. To increase tolerance to small deviations, a Gaussian weight centered at the true-bin index is introduced to weight the KL loss:

(23) $$\xi^{\prime }(i)=\sum_{c=1}^{C}w_{c}^{\left(i\right)}y_{c}^{\left(i\right)}\log\frac{y_{c}^{\left(i\right)}}{{\hat{y}}_{c}^{\left(i\right)}},\;\;\;w_{c}^{\left(i\right)}=\exp\left(-\frac{{\left(c-c^{*}\right)}^{2}}{2\sigma^{2}}\right)$$

In Equation (23), $c^{*}$ is the frequency-bin index corresponding to the ground truth heart rate, and σ controls the tolerance bandwidth (in the unit of frequency bins). For example, σ=3 corresponds to approximately 3 bins, i.e., 3×0.0122≈0.0366 Hz, which is on the order of ∼2.2 bpm under the resolution in Equation (23). This design assigns higher optimization weights to bins near the ground truth heart rate, thereby improving robustness of heart rate estimation across windows and states without sacrificing frequency resolution.

## 3. Construction of Inertial and Heart Rate Signal Recognition Methods

### 3.1. Passenger State Recognition Method Based on Inertial Thresholds

This section corresponds to the first stage of the proposed framework, namely kinematic risk screening based on inertial and physical quantity signals for rapid early warning. This section proposes a passenger state recognition method tailored for high-dynamic operational scenarios in amusement rides. The method integrates a standard threshold rule engine with a deep temporal sequence model to enable real-time state monitoring and anomaly detection. The core of this method is to obtain physical quantities such as acceleration, angular velocity, linear velocity, and attitude angles from IMU/SINS devices and automatically generate labeling rules based on the GB 8408-2018 standard. Then, a CNN-LSTM network is used to learn the temporal features of these physical quantities, enabling online state determination and safety alerts.

#### 3.1.1. System Architecture and Data Channel Design

**Figure 10:**
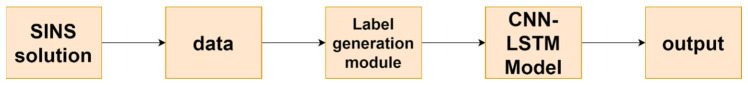
System design with multi-module collaboration.

As shown in Figure 10, the system adopts a multi-module collaborative architecture, forming a closed-loop process around “real-time physiological data acquisition—intelligent analysis—safety alerts.” The system uses a strapdown inertial navigation system (SINS) as the core for generating physical quantities: the inertial measurement unit (IMU) collects raw tri-axial accelerations and tri-axial angular velocities, and after processing through inertial navigation calculations, outputs tri-axial accelerations, tri-axial angular velocities, linear velocities, and attitude angles (Roll, Pitch, and Yaw). These physical quantities provide the data foundation for feature extraction, automatic labeling, and model training.

To ensure stable and reliable data, a preprocessing module is included in the system. The acceleration and angular velocity signals are processed using low-pass filters (cutoff frequency of 5 Hz) to suppress high-frequency noise and discrete spikes. For velocity and attitude angles, smoothing methods like sliding averages or exponential smoothing are used to reduce jitter and jumps. The continuous data stream is then segmented into sample units of fixed-length windows. Specifically, let the uniform sampling rate be $f_{s}$, and each sample contains T=200 frames. The window length is therefore $T/f_{s}$, and with T=200, this gives a window length of approximately 2 s when $f_{s}\approx 100\;\mathrm{Hz}$. In this setup, the input dimensions of a single sample are $\left(\mathrm{batch}\_\mathrm{size},200,12\right)$, where the 12 channels consist of four types of physical quantities: tri-axial acceleration ($A_{x},A_{y},{and\;A}_{z}$), tri-axial angular velocity ($\omega_{x},\omega_{y},{and\;\omega }_{z}$), tri-axial linear velocity ($V_{x},V_{y},{and\;V}_{z}$), and tri-axial attitude angles (ϕ,θ,and ψ) (corresponding to Roll, Pitch, and Yaw).

As shown in Table 1, the meanings and roles of each physical quantity are as follows: Acceleration is used to characterize the linear impacts on passengers in different directions, suitable for identifying abrupt accelerations and sudden motions. Angular velocity describes the intensity of rotation and changes in direction, suitable for detecting rotational anomalies. Linear velocity reflects the speed of movement relative to the ground. Attitude angles describe the tilt and rotation of the passenger coordinate system. To enhance engineering reliability, dual-IMU redundancy or external references (e.g., RTK) can be used to improve fault tolerance and robustness, but this section uses the unified 12-channel physical quantity sequence as input.

The core model employs a CNN-LSTM hybrid structure for state recognition. The network takes the physical quantity sequence $X\in R^{200\times 12}$ as input and applies convolutional layers to extract short-term local anomaly patterns. An LSTM network is then used to model the temporal dependencies, ultimately outputting three state probabilities $\hat{p}=[{\hat{p}}_{0},{\hat{p}}_{1},{\hat{p}}_{2}]$ (normal/mild anomaly/high risk). When the probabilities for anomalies or high risk exceed preset thresholds, the system triggers safety strategies, such as audible alarms, emergency braking, and remote notifications, thus enabling data-driven intelligent safety management.

#### 3.1.2. Automatic Label Generation Based on Standards

The GB 8408-2018 standard (safety requirements for large amusement facilities) provides safety thresholds and testing requirements for physical quantities during ride operations. It focuses on parameters such as acceleration, angular velocity/angular acceleration, velocity, and shock rate, which help reduce risks such as dizziness, impact injuries, and structural/system failures. Examples of relevant thresholds are shown in Table 2 (e.g., peak acceleration, synthesized acceleration, peak angular velocity, peak angular acceleration, emergency braking deceleration limits, etc.). It is important to note that the tests in the standard mainly target type tests and safety verification scenarios. In this study, we aim for online monitoring and algorithm training. Given the sensor precision and time synchronization, we use fixed sampling rates $f_{s}$ for the physical quantity sequences and enhance the capture ability of peaks and short-term anomalies via windowed statistics and contextual methods.

To construct high-quality supervised samples, the system is designed with an automatic label generation algorithm based on standard thresholds. For each window of 200 frames (2 s), statistical features such as peaks, root mean square (RMS), duration, and rate of change are extracted. Typical feature definitions are as follows (using acceleration as an example):

Synthetic Acceleration Peak:

(24) $$A_{\mathrm{mag},\;\max}=\max_{t}\sqrt{A_{x}{\left(t\right)}^{2}+A_{y}{\left(t\right)}^{2}+A_{z}{\left(t\right)}^{2}}$$

Exceeding Threshold Duration (for threshold τ):

(25) $$\mathrm{duration}(\tau )=\frac{1}{f_{s}}\sum_{t=1}^{200}I(A_{z}(t)>\tau )$$

Attitude Rate of Change (using Pitch as an example, approximated by differentiation):

(26) $$\dot{\theta }(t)\approx \frac{\theta (t)-\theta (t-1)}{1/f_{s}},{\dot{\theta }}_{\max}=\max_{t}|\dot{\theta }(t)|\;$$

In label design, a three-level classification is used to balance interpretability and risk management requirements: normal (0), mild anomaly (1), and high risk (2). The priority logic for classification follows “high risk first, then mild anomaly,” and aligns with the thresholds shown in Table 2.

To construct high-quality supervised learning samples, the system is designed with an automatic labeling algorithm based on national standards. This algorithm extracts statistical features of various physical quantities for each fixed-length time window (e.g., 200 frames, 2 s) and applies the safety thresholds for acceleration, angular velocity, velocity, and attitude angles in GB 8408-2018 to implement sample auto-labeling.

The specific method includes extracting the maximum value, mean, and rate of change of acceleration (Ax, Ay, and Az), angular velocity (Gx, Gy, and Gz), linear velocity (Vx, Vy, and Vz), and attitude angles (Roll, Pitch, and Yaw). The focus is on the peak value of Az and its duration above the threshold, sudden increases in lateral acceleration, rapid rotation of Gz, and the rate of change in Pitch/Roll (calculated through frame differences). These features are used to determine whether a sample has potential risks.

As shown in Algorithm 1, the system employs a hierarchical judgment strategy: if Az_max exceeds 3.5 g and lasts more than 2 s, it is labeled as high risk (label = 2); if Ax_max exceeds 2 g, Gz_max exceeds 6 rad/s, or the Pitch/Roll rate of change exceeds 30°/s, it is labeled as a mild anomaly (label = 1); otherwise, it is labeled as normal (label = 0). The algorithm supports dynamic sampling frequency conversion and sliding window statistics to improve timeliness and accuracy.

To reduce false positives, the system introduces smoothing techniques such as median filtering and sliding average and uses a context window voting mechanism (e.g., if two out of three consecutive windows show anomalies, it is marked as anomalous). This enhances the stability and robustness of the labels.

The entire labeling process can run automatically and output structured data formats (such as .csv or .npy), making it easy for subsequent model training. This mechanism effectively ensures the consistency and standardization of labeling, significantly reducing labor costs and serving as an important support module for building dynamic threshold-enhanced models.

It should be noted that the automatically generated labels in this study are intended as standards-guided engineering supervision rather than definitive expert ground truth. Although this strategy improves interpretability and reproducibility under explicit safety constraints, dedicated expert validation and threshold sensitivity analysis remains to be conducted in future work.
**Algorithm** **1:** Decision rules for physical quantity based state labelsInput: Statistical characteristics of physical quantities within a time window:Az_max, Ax_max, Gz_max, pitch_rate, durationOutput: Status label (0 = normal, 1 = mildly abnormal, 2 = high risk)If Az_max > 3.5 and duration > 2.0 s:        Return to label 2 (high risk)Otherwise if Ax_max > 2.0 or Gz_max > 6.0 or |pitch_rate| > 30°/s:        Return label 1 (mild abnormality)end if:        Return label 0 (normal)

#### 3.1.3. Dynamic Threshold Model Design and Training

Threshold rules provide highly interpretable initial classification, but their applicability is limited in the presence of sensor noise, individual differences, and complex dynamic patterns. To further enhance the recognition ability for “critical risks,” “composite anomaly patterns,” and “time-dependent features,” this section adopts a CNN-LSTM structure to learn anomaly patterns in the physical quantity time series, achieving dynamic threshold enhancement via model fitting and generalization.

(1) Model Input and Structure

The training data consists of 12-channel physical quantity sequences obtained from SINS calculations. The input dimensions are $\left(\mathrm{batch}\_\mathrm{size},200,12\right)$, where 200 is the time-frame length, and 12 refers to the number of channels corresponding to acceleration, angular velocity, linear velocity, and attitude angles. The output labels are generated by the automatic rule engine in Section 3.1.2 and consist of three categories: normal (0), mild anomaly (1), and high risk (2).

The network structure and parameter configuration are as shown in Table 3: Conv1D(64) → MaxPooling → Conv1D(128) → MaxPooling→LSTM(64) → Dense(32) → Dense(3, Softmax).

In this structure, convolutional layers capture short-term local anomalies and patterns (such as spikes, oscillations, and abrupt increases/decreases). The LSTM layer models the temporal dependencies, while the final Softmax output provides a probability distribution over the three states.

(2) Training Strategy and Loss Function

Before training, each channel is normalized to eliminate dimensionality effects. After shuffling, the dataset is divided into training, validation, and test sets (e.g., 70%/10%/20%). The training objective is to minimize the categorical cross-entropy loss:

(27) $$L=-\sum_{c=0}^{2}y_{c}\log{\hat{p}}_{c}\;$$

The Adam optimizer is used with an initial learning rate of $1\times 10^{-3}$. During training, performance is monitored using metrics such as accuracy, precision, recall, and F1-score, with a special focus on the recall of anomaly and high-risk categories to reduce missed detections and ensure safety.

The model adopts mini-batch stochastic gradient descent (e.g., batch size = 32) with a maximum of 100 epochs and early stopping to prevent overfitting (e.g., stop if validation loss does not improve for 10 consecutive epochs). For robustness, lightweight data augmentation (e.g., adding small noise to some channels, simulating attitude drift or short-term impact disturbances) is applied to improve model adaptability to sensor noise and varying conditions.

After training, the model is evaluated on the test set using metrics such as confusion matrix, precision/recall/F1-score, and average inference latency. These evaluations guide deployment on edge devices for real-time safety alerts.

Although inertial signals provide crucial information for passenger state recognition, physiological responses (such as heart rate, blood oxygen levels, etc.) also play a vital role in complex, high-dynamic environments. Therefore, based on the analysis of inertial signals, we further incorporate physiological signals for state classification and diagnosis to improve the accuracy and reliability of state recognition. The following section will introduce the passenger state diagnosis method based on physiological signals, enhancing the capability of state determination.

### 3.2. Passenger State Classification and Diagnosis Based on Physiological Signals

This section corresponds to the second stage of the proposed framework, namely physiological diagnosis based on physiological signals. Unlike the first stage abnormal state screening task, which is intended for rapid warning, the second stage task focuses on finer-grained interpretation of abnormal segments and rhythm-related physiological classification. In Section 3.1, abnormal passenger states were preliminarily identified using inertial signals and physical quantity thresholds, enabling rapid detection of potentially unsafe dynamic conditions. However, under high-dynamic amusement ride environments, physiological responses such as heart rate variation and rhythm abnormality provide more direct evidence of the passenger’s internal state. Therefore, this section introduces a physiological-signal-based diagnosis module to perform finer-grained classification and interpretation of abnormal segments. In this way, the overall framework forms a two-stage closed-loop: rapid warning based on physical quantities in the front end, followed by physiological diagnosis in the back end.

#### 3.2.1. Physiological Signal Diagnosis Model Design

High-dynamic conditions such as rapid acceleration, vibration, posture change, and psychological stress make physiological signals highly non-stationary and noise-contaminated. To address this problem, this study develops a deep physiological diagnosis model for abnormal segment interpretation. The adopted architecture is motivated by the characteristics of high-dynamic physiological monitoring rather than by a simple combination of standard modules. Residual convolution is used to preserve local waveform morphology under noise contamination, BiLSTM is used to model temporal dependency in both directions, and multi-head attention is introduced to emphasize diagnostically informative temporal regions in non-stationary sequences.

To align with the early warning logic in Section 3.1, this section adopts a two-stage task definition with unified label semantics. Phase I corresponds to online monitoring, in which short-window segments are classified as normal or abnormal for rapid warning. Phase II is triggered only for abnormal segments and focuses on fine-grained physiological diagnosis. In this study, the diagnostic task targets six rhythm-related categories, including SR, PVC, PAC, VT, SVT, and AF.

**Figure 11:**
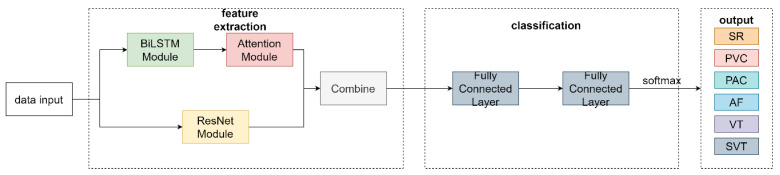
HS-BANet Framework.

For this purpose, a parallel spatiotemporal feature modeling framework, HS-BANet, is proposed, as shown in Figure 11. The model consists of a spatial branch and a temporal branch. The spatial branch uses an improved one-dimensional residual network to extract local waveform morphology and discriminative short-range features from physiological signals. The temporal branch uses stacked BiLSTM layers to capture bidirectional temporal dependency and a multi-head self-attention module to adaptively focus on salient temporal segments. The outputs of the two branches are concatenated and passed through fully connected layers to generate the final classification result.

(1) Spatial Feature Extraction Module

**Figure 12:**
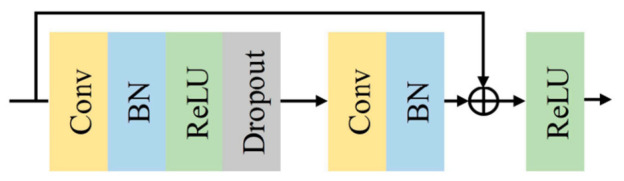
Optimized ResNet structure.

The spatial feature extraction branch is based on a one-dimensional residual network, as shown in Figure 12. Compared with conventional convolutional stacking, residual connections help preserve useful low-level features and improve training stability in noisy physiological signals. In the present model, the residual branch is used mainly to extract local morphology and short-range discriminative patterns from the input waveform. To further improve convergence and reduce overfitting, batch normalization is used after convolution, and dropout is introduced after nonlinear activation. The resulting feature representation provides robust local descriptors for subsequent temporal modeling.

(2) Time Feature Extraction Module

**Figure 13:**
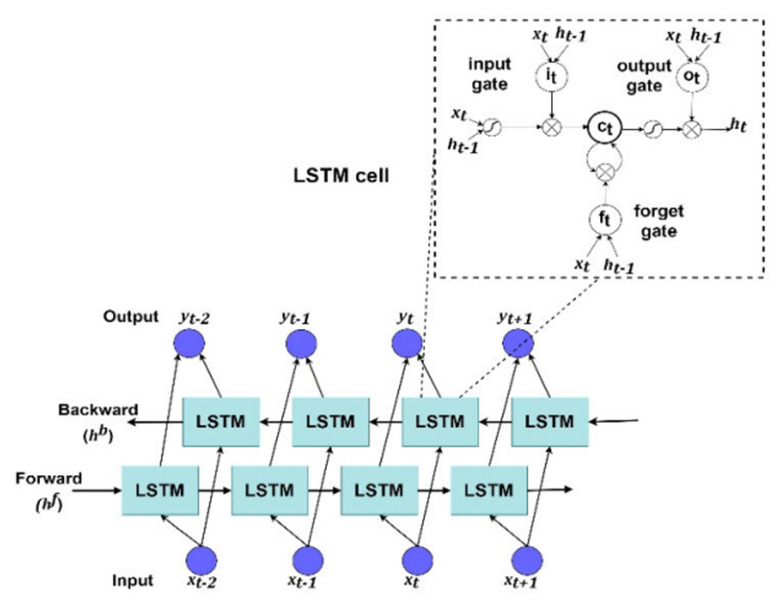
Overall structure of BiLSTM.

To capture long-range temporal dependency in physiological sequences, a BiLSTM-based temporal modeling branch is adopted, as shown in Figure 13. Unlike unidirectional recurrent modeling, BiLSTM can simultaneously incorporate forward and backward contextual information, which is particularly useful for physiological sequences with strong temporal dependency and local irregularity. In this study, a four-layer BiLSTM structure with 128 hidden units per layer is used to encode temporal dynamics.

To further enhance the discriminative ability of the sequence representation, a multi-head self-attention mechanism is applied after the BiLSTM output. Instead of providing a general tutorial-style description, only the task-relevant formulation is retained here. The attention output is computed as:

(28) $$\mathrm{Attention}(Q,K,V)=\mathrm{Softmax}\left(\frac{QK^{T}}{\sqrt{d_{k}}}\right)V$$

where Q, K, and V represent the query, key, and value vector sets, respectively, and $d_{k}$ is the dimension of the key vectors.

**Figure 14:**
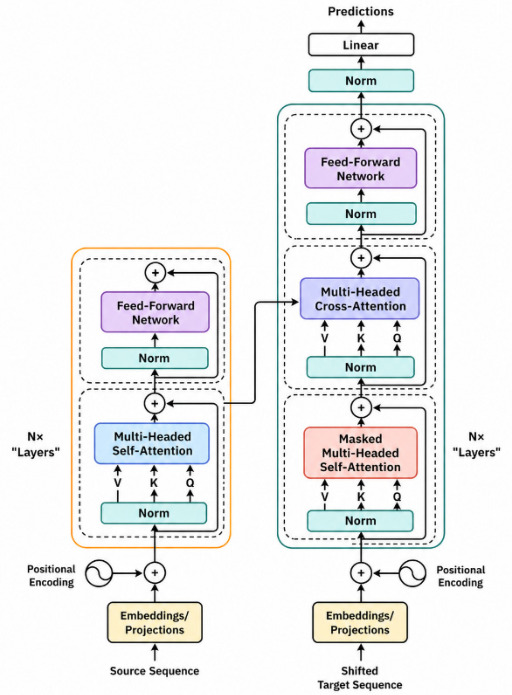
Schematic illustration of the multi-head self-attention mechanism used in the temporal feature extraction branch, following the standard formulation in [50].

The multi-head self-attention mechanism adopted here follows the standard formulation introduced in prior work [49] and is used in this study as a task-specific temporal feature refinement module. In Figure 14, in multi-head self-attention, the model executes multiple independent attention computations in parallel, with each attention head focusing on different subspace representations. The outputs from each head are concatenated and mapped back to the original dimension via a learnable linear transformation $O_{W}$:

(29) $$\mathrm{MultiHeadAttention}(Q,K,V)=\mathrm{Concat}({\mathrm{head}}_{1},\ldots ,{\mathrm{head}}_{h})W^{O}$$

where ${\mathrm{head}}_{i}$ denotes the output from the i-th attention head, and $W_{O}$ is the output mapping matrix.

By incorporating multi-head self-attention after the BiLSTM output, the model can not only capture broader global dependencies but also focus on specific time segments, effectively reducing noise and enhancing feature expression. Compared to the sequential information transmission method of RNNs, this parallel strategy offers significant advantages in computational efficiency and feature integration, laying a solid foundation for subsequent classification tasks.

#### 3.2.2. Experimental Setup and Evaluation Metrics

All physiological diagnosis experiments were implemented using PyTorch 2.0.0 and Python 3.10. Model training and evaluation were conducted on a workstation equipped with an NVIDIA GeForce RTX 3090 GPU, an Intel Core i9-12900K processor, and Ubuntu 22.04 LTS. This configuration provided sufficient computational support for multi-class physiological time series training and comparison.

For model development, the dataset was randomly shuffled and then divided into training, validation, and test sets at a ratio of 6:2:2, yielding 28,097, 9365, and 9365 samples, respectively. During training, cross-entropy loss was used as the optimization objective, and model parameters were updated through backpropagation. An adaptive learning-rate decay strategy was adopted, and early stopping was used to reduce overfitting and unnecessary computation.

To evaluate classification performance, standard multi-class metrics were used, including accuracy, precision, recall (sensitivity), specificity, and F1-score. These metrics were jointly used to assess the model from the perspectives of overall accuracy, abnormal-class detection capability, false-positive control, and class-imbalance robustness. For the physiological diagnosis task, in addition to convolution-based deep learning baselines, a BiLSTM model was further implemented as a sequence-aware comparator for physiological time series classification.

By combining physiological and inertial signals, the proposed framework not only enhances the system’s ability to detect short-term sudden anomalies but also improves its sensitivity to longer-term physiological changes. This multimodal design provides practical support for passenger health monitoring and safety warning in high-dynamic amusement ride environments.

## 4. Results and Discussion

During the operation of large-scale amusement rides, passengers are exposed to complex dynamic conditions—such as impulsive acceleration shocks, rotational motion, and rapid attitude changes—which may induce diverse physiological abnormalities and, in severe cases, pose health risks. Therefore, developing a reliable and efficient intelligent diagnostic framework is essential for enhancing rider safety and improving operational management. In this chapter, leveraging the physical quantities provided by a strapdown inertial navigation system (SINS) and the Chinese national standard GB 8408-2018, we establish a real-time, broadly applicable threshold-based recognition mechanism for kinematic variables, and further design a CNN–LSTM dynamic threshold-enhanced model (TAPNet) to enable accurate identification and early warning of abnormal passenger states. For physiological signals, we propose HS-BANet, which integrates a convolutional neural network with a bidirectional long short-term memory network (BiLSTM) to deeply mine discriminative patterns from multi-channel time series data, including heart rate, electrodermal activity, and acceleration, thereby effectively recognizing complex conditions such as tension, dizziness, and loss of consciousness. Comprehensive evaluations and comparative analyses demonstrate that the proposed methods achieve superior performance in terms of accuracy, robustness, and practical deployability.

### 4.1. Heart Rate Estimation Results and Comparative Analysis

Under high-dynamic motion conditions, photoplethysmography (PPG) signals are highly susceptible to vigorous body movements, variations in contact pressure, and tissue deformation, which introduce pronounced motion artifacts and non-stationary noise. As a result, conventional heart rate estimation methods often suffer from spectral-peak drift, abrupt estimation jumps, or discontinuous tracking trajectories during moderate-to-high intensity activities. To address these challenges, this study develops a deep learning–based heart rate estimation model and conducts systematic evaluations across multiple datasets. This section first presents the implementation details, training protocol, and evaluation metrics, and then benchmarks the proposed approach against several representative heart rate estimation methods. The results substantiate the superiority of the proposed model in terms of accuracy, temporal stability, and cross-subject generalization, with particular emphasis on its robustness under severe motion artifact conditions.

#### 4.1.1. Experimental Implementation and Training Strategy

The proposed heart rate estimation model was implemented in **PyTorch 2.1.0** and trained/evaluated under a standardized workflow using **PyTorch Lightning 2.1.2**. **NumPy 1.26.4** was employed for data organization and statistical computation, while **Matplotlib 3.8.2** was used to visualize training curves and experimental results. The model was trained using the **AdamW optimizer implemented in PyTorch**, with a learning rate of $1\times 10^{-4}$ and a weight-decay coefficient of $1\times 10^{-4}$. The batch size was set to 8, and training was accelerated using an **NVIDIA GeForce RTX 3090 GPU with 24 GB memory** [NVIDIA GeForce RTX 3090 GPU (ASUSTeK Computer Inc., Taipei, Taiwan)]. To mitigate gradient explosion, gradient clipping was applied with a clipping threshold of 1.0, which stabilized convergence. To mitigate gradient explosion, gradient clipping was applied with a clipping threshold of 1.0, which stabilized convergence.

To comprehensively assess the model’s generalization capability for heart rate estimation, five-fold cross-validation was conducted on HSSH-I (augmented training set) for model selection and hyperparameter tuning. Notably, because samples were constructed via a sliding window scheme (e.g., an 8 s window length with a 2 s step size), substantial overlap exists between adjacent windows. A naive random split at the window level would therefore introduce information leakage between training and validation sets. To avoid this issue, we adopted a subject-wise Group K-Fold strategy, ensuring that data from the same subject never appeared in both the training and validation folds, thus guaranteeing an unbiased evaluation. In each fold, the model was trained on the training split, and the best epoch was selected based on the average validation loss on the validation split. The final model was then trained on the full HSSH-I dataset using the optimal configuration.

For final evaluation, a one-shot independent test was performed on HSSH-II (independent validation set) to examine the model’s practical cross-subject generalization performance.

The primary accuracy metric was the root mean square error (RMSE, in bpm). In addition, correlation and agreement analyses were reported: the Pearson correlation coefficient r was used to quantify the linear association between the estimated and reference heart rates, and Bland–Altman analysis was conducted to provide the mean bias and limits of agreement (LOA), enabling assessment of systematic bias and stability across different heart rate ranges.

#### 4.1.2. Comparison Results with Mainstream Methods

To evaluate the accuracy of the proposed approach for dynamic heart rate estimation, we compared RMSE against several representative methods on both HSSH-I and HSSH-II. The compared baselines include a PPG-only estimator (PPG only), weighted frequency-peak voting (WFPV), frequency spectrum method (FSM), kernel-enhanced frequency spectrum method (Kernel-FSM), sparse spectrum method (SPF), and multi-spectrum method (MPF). The proposed approach is denoted as “Proposed Model.”

On HSSH-I (n=25), the conventional PPG-only method yielded the largest error, with an average RMSE of 16.31 bpm. WFPV achieved an average RMSE of 8.75 bpm. The average RMSEs of FSM, Kernel-FSM, SPF, and MPF were 3.04 bpm, 2.12 bpm, 7.06 bpm, and 3.33 bpm, respectively. In contrast, the proposed model achieved an average RMSE of only 1.18 bpm across all subjects, with multiple subjects exhibiting errors below 1 bpm. These results indicate that the proposed approach maintains high accuracy and stability even under severe motion artifacts and high-dynamic perturbations.

On HSSH-II (n=23), the overall trend remained consistent with that observed on HSSH-I. The average RMSEs of the PPG-only method and WFPV were 7.77 bpm and 4.72 bpm, respectively. MPF achieved an average RMSE of 3.00 bpm, while FSM and Kernel-FSM achieved 1.49 bpm and 2.04 bpm, respectively. The proposed model again delivered the best performance, with an average RMSE of 1.24 bpm. Notably, for several low-SNR samples, the proposed method still maintained RMSE within the 1–1.5 bpm range, demonstrating strong robustness and cross-subject generalization.

**Figure 15:**
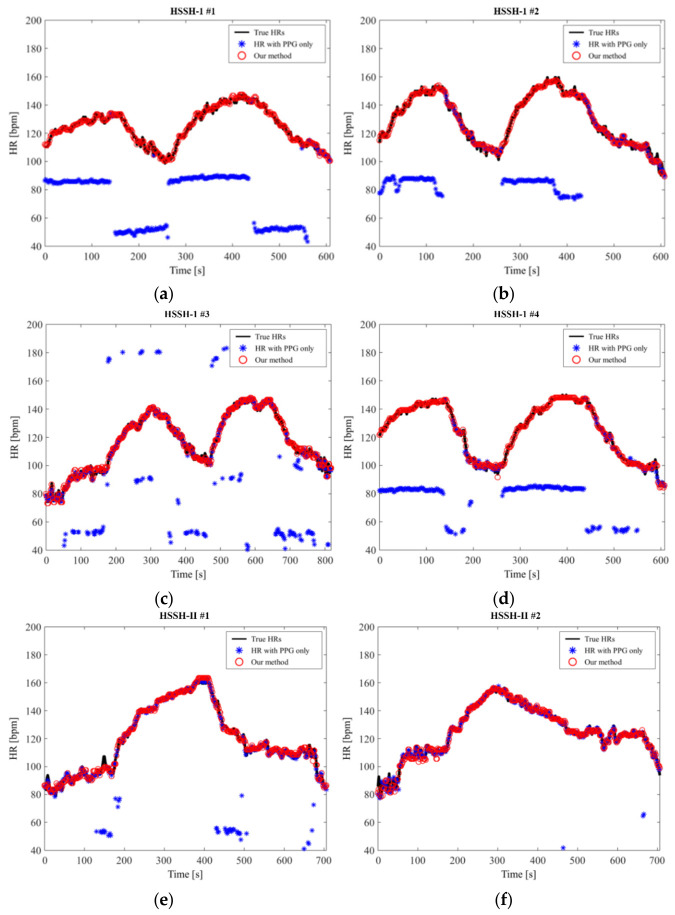
Heart rate estimation. (**a**) Results of Subject 1 on the HSSH-I dataset; (**b**) results of Subject 2 on the HSSH-I dataset; (**c**) results of Subject 3 on the HSSH-I dataset (**d**) results of Subject 4 on the HSSH-I dataset; (**e**) results of Subject 1 on the HSSH-II dataset; and (**f**) results of Subject 2 on the HSSH-II dataset.

Figure 15 visualizes heart rate tracking trajectories for different subjects under high-dynamic conditions and illustrates the relationship between the estimated heart rate and changes in acceleration intensity. Specifically, Figure 15a–d present representative subjects from HSSH-I, while Figure 15e,f show representative results from the independent HSSH-II test set. It can be observed that the PPG-only method is more prone to fluctuations, abrupt jumps, or pronounced deviations during moderate-to-high intensity motion. By comparison, the proposed method remains more closely aligned with the reference heart rate curve across different motion phases, enabling stable tracking of rapid increases, oscillations, and recovery dynamics—thereby evidencing stronger resistance to motion artifacts and improved trajectory continuity.

**Figure 16:**
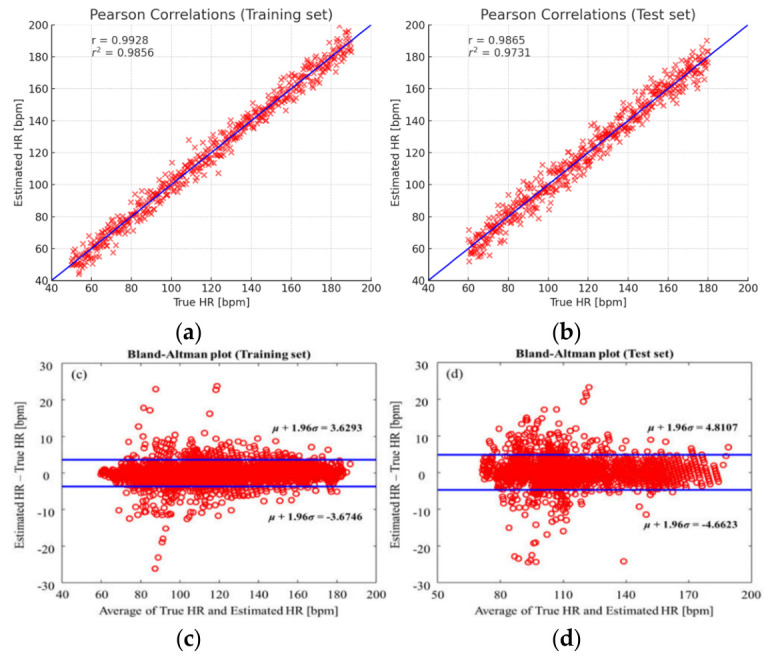
Analysis of correlation and consistency between estimated heart rate and true heart rate. (**a**) Pearson correlation coefficient of the training set (r = 0.9928); (**b**) Pearson correlation coefficient of the test set (r = 0.9865); (**c**) training set Bland–Altman plot; (**d**) test set Bland–Altman plot.

Figure 16 presents the Pearson correlation analysis and Bland–Altman visualizations between the estimated heart rate (HR) and the ground truth HR on both the training and test datasets. As shown in Figure 16a,b, the Spearman rank correlation coefficients of the proposed model are 0.9928 $\left(\rho^{2}=0.9856\right)$ and 0.9865 $\left(\rho^{2}=0.9731\right)$ for the training and test datasets, respectively.

Figure 16c,d depict the Bland–Altman plots for each dataset. For the training dataset, the limits of agreement (LOA) range from −3.72 bpm to 3.58 bpm (mean bias: 0.03 bpm; standard deviation: 1.89 bpm). Similarly, for the test dataset, the LOA ranges from −4.72 bpm to 4.85 bpm (mean bias: 0.08 bpm; standard deviation: 2.45 bpm).

Through an in-depth analysis of the heart rate estimation results, the proposed approach demonstrates superior accuracy and robustness in dynamic environments, maintaining high estimation fidelity even under pronounced motion artifact interference. In the following, this chapter shifts to passenger state recognition based on physical quantity thresholds, further investigating how abnormal passenger conditions can be effectively identified in complex high-dynamic scenarios and how the developed models can be leveraged for timely warning and risk mitigation.

### 4.2. State Recognition Results Based on Physical Quantity Thresholds

This section provides an in-depth investigation of the state recognition capability enabled by the physical quantity threshold rules proposed in Section 3 and the dynamic-threshold-enhanced model TAPNet. By modeling the temporal characteristics of multi-channel kinematic variables, TAPNet effectively identifies abnormal behaviors across different passenger states and is further benchmarked against several conventional classifiers. Experimental results demonstrate that TAPNet achieves clear improvements in both recognition accuracy and recall for abnormal states and exhibits strong robustness when handling class-imbalanced data, underscoring its practicality for reliable detection in high-dynamic scenarios.

#### 4.2.1. Model Evaluation and Experimental Analysis

After the preprocessing and attitude determination procedures described in Section 3, the sampling frequency was set to 100 Hz. Each sample was constructed using a fixed-length window of 200 frames, i.e., 2 s per sample. Following data cleaning, window segmentation, and automatic labeling, 2370 valid motion segments were retained. Based on the safety thresholds specified in GB 8408-2018, the resulting windows were categorized into two classes: normal and abnormal. Specifically, the dataset contains 45,352 normal samples and 3266 abnormal samples. This distribution indicates a pronounced class imbalance: normal samples account for the vast majority (approximately 93.3%), whereas abnormal samples represent only 6.7%, which is consistent with real-world operations where abnormal states occur relatively infrequently. Despite the imbalance, the two classes exhibit clear separability in terms of signal magnitude, rate of change, and duration-related characteristics, which facilitates the learning of discriminative patterns and supports accurate abnormal state recognition—thereby providing a solid data foundation for subsequent model training.

During model training, the proposed TAPNet employs a CNN–LSTM architecture to model and classify the temporal dynamics of multi-channel physical quantities. The input is a tensor of shape $\left(200\times 12\right)$, where 200 denotes the time-window length and 12 corresponds to the channels of tri-axial acceleration, angular velocity, velocity, and attitude. The feature-extraction stage consists of two 1D convolutional layers with pooling, designed to capture local transients and frequency-related patterns. A single LSTM layer is then used to model temporal dependencies, followed by fully connected layers and a Softmax classifier to output the final state label.

For optimization, TAPNet is trained using the Adam optimizer with an initial learning rate of $1\times 10^{-3}$, a batch size of 32, and a maximum of 100 epochs. To mitigate overfitting, early stopping is applied with a patience of 10 epochs based on the validation loss. Labels are generated by the aforementioned GB-standard-based automatic labeling module, and the dataset is split into 70%/10%/20% for training, validation, and testing, respectively. For performance benchmarking, several classical classifiers—including Random Forest, Support Vector Machine, and Decision Tree—are also evaluated. The detailed hyperparameter settings for all models are provided in Table 4.

#### 4.2.2. Model Comparison and Analysis

**Figure 17:**
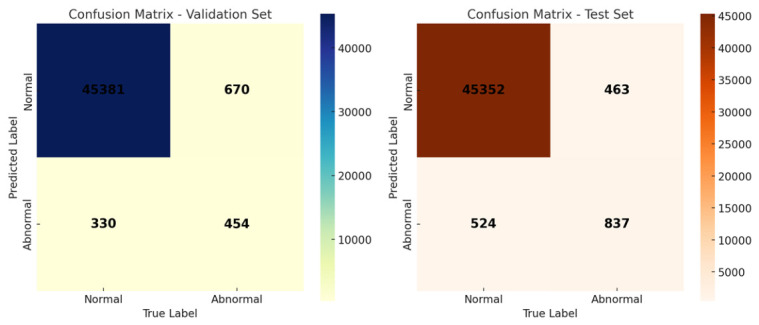
Confusion matrix of the TAPNet model on the validation set and test set.

After training the proposed TAPNet (Threshold-Aware Physical Network) on the same dataset, its performance was evaluated on both the validation and test sets. TAPNet achieved an accuracy of 96.9% on the validation set, which further increased to 98.2% on the test set. The corresponding confusion matrices are shown in Figure 17.

As can be observed, on the validation set, the model correctly identified 454 abnormal samples while misclassifying 670 abnormal instances as normal. It also produced 330 false positives, i.e., normal samples incorrectly predicted as abnormal, and correctly classified 45,381 normal samples. On the test set, TAPNet correctly detected 837 abnormal samples and misclassified 524 abnormal samples as normal; meanwhile, 463 normal samples were incorrectly predicted as abnormal, and 45,352 normal samples were correctly recognized.

Overall, TAPNet maintains consistently high recognition performance on both splits, demonstrating strong capability in detecting abnormal states while preserving a high classification accuracy for normal states—indicative of robust generalization and practical reliability. Compared with conventional methods, TAPNet substantially improves the recall of abnormal conditions and the overall accuracy. Moreover, its low-latency and lightweight design makes it a promising candidate for real-world deployment on embedded platforms.

**Figure 18:**
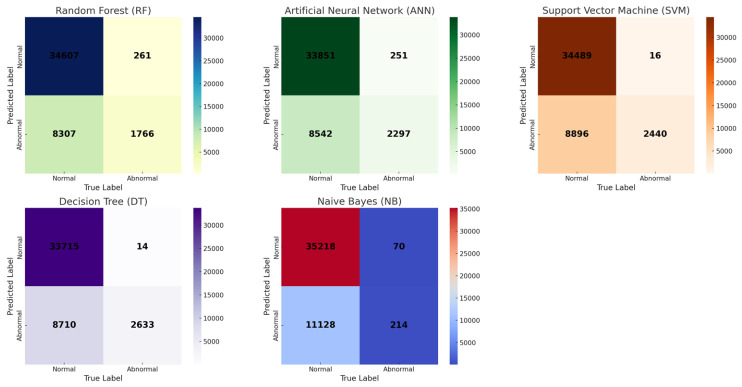
Confusion matrix performance of various traditional classification models on validation and test sets.

To evaluate the performance of conventional classifiers for passenger state recognition, five representative machine-learning methods were introduced for comparative experiments: Random Forest (RF), Artificial Neural Network (ANN), Support Vector Machine (SVM), Decision Tree (DT), and Naive Bayes (NB). All models were trained using the same feature inputs, with the recognition target defined as a binary label (normal/abnormal). Their corresponding confusion matrices are presented in Figure 18. The training data were collected from 46 randomly selected participants, whereas the validation and test sets were drawn from an additional 25 independent subjects. The data split protocol was kept identical to that used for TAPNet to ensure a fair comparison.

As shown in Figure 18, except for RF, the other models did not incorporate explicit class-balancing strategies during training, which led to consistently weak recognition performance for the minority (abnormal) class. Specifically, RF and ANN exhibited relatively balanced overall behavior: both achieved high prediction accuracy for normal samples and comparatively stronger capability in detecting abnormal states, correctly identifying 1766 and 2297 abnormal samples, respectively. However, both still suffered from non-negligible missed detections—RF misclassified 8307 abnormal samples as normal, while ANN misclassified 8542.

Although SVM produced almost no errors for the normal class (only 16 normal samples were incorrectly predicted), its ability to recognize abnormal conditions was clearly insufficient. It correctly detected only 2440 abnormal samples, while misclassifying the remaining 8896 as normal, indicating severe bias toward the majority class. The DT model was relatively stable for normal-state recognition but remained limited in abnormal detection: it correctly identified 2633 abnormal samples and misclassified 8710, suggesting susceptibility to overfitting in complex scenarios. Overall, NB delivered the weakest performance, correctly detecting only 214 abnormal samples and assigning 11,128 abnormal instances to the normal class, demonstrating clearly inadequate accuracy and robustness.

**Figure 19:**
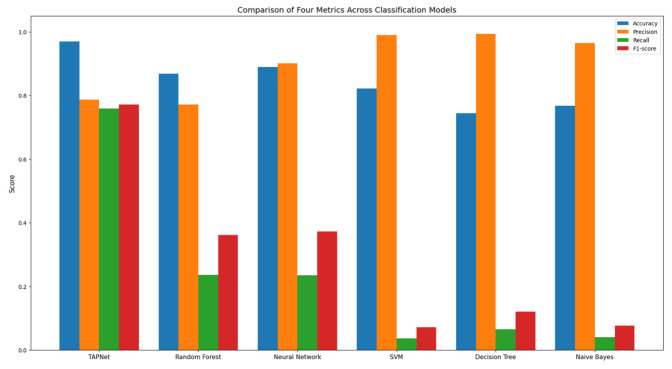
Comparative analysis of performance metrics of various classification models.

As shown in Figure 19, a comparative evaluation of six classification models is reported using four standard performance metrics for the passenger state recognition task: accuracy, precision, recall, and F1-score. The horizontal axis lists the evaluated models—TAPNet, Random Forest, Artificial Neural Network, Support Vector Machine, Decision Tree, and Naive Bayes—while the vertical axis indicates the metric scores ranging from 0 to 1.

Overall, TAPNet achieves the best performance across all four metrics: its accuracy is close to 1, and its precision, recall, and F1-score also remain consistently high, highlighting its clear advantage in balancing the recognition of normal and abnormal states. In comparison, Random Forest and ANN exhibit relatively balanced behavior with high accuracy and precision; however, their recall and F1-score are noticeably lower than those of TAPNet, indicating that their ability to detect abnormal states remains limited.

The performance gaps among SVM, DT, and NB are more pronounced. Although these models achieve comparable—or even relatively high—precision, their recall and F1-score are extremely low. This is particularly evident for Naive Bayes and SVM, whose recall values fall below 0.1, suggesting a severe deficiency in detecting abnormal samples and a clear majority-class bias that leads to overall model imbalance.

Taken together, Figure 19 provides an intuitive visualization of the performance disparities among competing methods under class imbalance and multi-channel dynamic time series conditions, further substantiating the comprehensive superiority and practical deployment potential of TAPNet for this task.

Although the physical quantity threshold–based recognition approach can effectively identify abnormal states, passengers’ physiological responses—such as heart rate variations and electrodermal activity—provide particularly critical evidence for state inference in highly dynamic environments. Therefore, building upon the results in Section 4.2, this section further introduces a physiological-signal–driven framework for state classification and diagnosis. By leveraging deep learning models to exploit multi-channel physiological time series patterns, the proposed approach aims to further improve the accuracy and reliability of abnormal state recognition.

### 4.3. State Classification and Diagnostic Results Based on Physiological Signals

In Section 4.2, abnormal passenger states were preliminarily categorized and identified using physical quantity thresholding. However, fine-grained diagnosis of physiological conditions—such as heart rate abnormalities, tension, or dizziness—requires dedicated analysis of physiological signals. To this end, this section proposes a PPG-based deep learning model, HS-BANet, which integrates a convolutional neural network (CNN), a bidirectional long short-term memory network (BiLSTM), and a multi-head self-attention mechanism to further extract discriminative abnormal patterns from physiological signals, enabling more effective and reliable abnormal state diagnosis.

**Figure 20:**
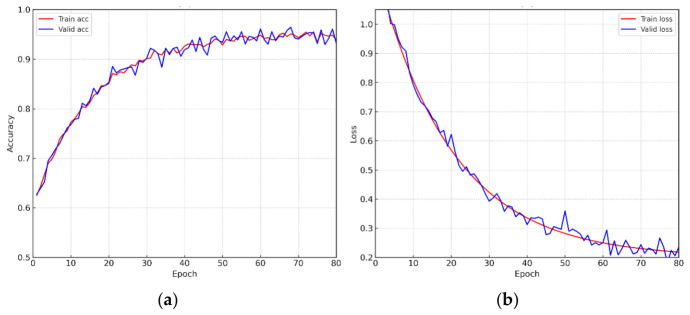
Accuracy and loss curves during model training. (**a**) Accuracy curve, (**b**) loss curve.

For model development, the dataset was first randomly shuffled and then split into training, validation, and test sets at a 6:2:2 ratio, yielding 28,097, 9365, and 9365 samples, respectively. During training, cross-entropy was used as the loss function, and network parameters were optimized via backpropagation. As illustrated in Figure 20, the initial learning rate was set to a predefined value and gradually decayed as training progressed. Once the validation loss failed to decrease for several consecutive epochs, an early-stopping mechanism was triggered to prevent overfitting and reduce unnecessary computation.

After training, the model was evaluated on the test set using a comprehensive set of metrics, including classification accuracy, precision, recall, specificity, sensitivity, and F1-score, so as to assess its recognition performance across different rhythm categories from multiple perspectives. As summarized in Table 5, the model achieves overall strong performance in discriminating among the six arrhythmia types, while a small number of misclassifications remain. For instance, approximately 2.1% of normal sinus rhythm (SR) samples are incorrectly classified as premature ventricular contractions (PVC), and 6.35% of premature atrial contractions (PAC) are misidentified as PVC. In addition, 3.63% of SR samples are mistakenly predicted as supraventricular tachycardia (SVT), whereas 7.11% of SVT samples are assigned to other categories.

When examining the learning curves of the loss and accuracy throughout training, we observe that the loss decreases progressively and then stabilizes, while the accuracy continues to increase and eventually converges. Notably, by incorporating adaptive learning-rate decay and early stopping, overfitting is substantially mitigated, and the validation accuracy becomes more stable across epochs.

A class-wise evaluation further reveals heterogeneous recognition performance across rhythm categories, including normal sinus rhythm (SR), premature atrial contractions (PAC), premature ventricular contractions (PVC), ventricular tachycardia (VCT/VT), supraventricular tachycardia (SVT), and atrial fibrillation (AF). Among them, AF exhibits the most prominent performance, achieving an average accuracy of 98.4%, with consistently high precision, recall, and F1-score. In contrast, although the overall detection rate for VT exceeds 90%, non-negligible misclassifications persist, suggesting that the model still faces challenges in distinguishing certain rhythm types with similar morphological patterns.

Overall, the model attains a six-class rhythm recognition accuracy of 91.38%, with a mean precision and recall of 86.5% and 83.5%, respectively, and an average F1-score of approximately 84.9%. The mean specificity reaches 96.4%, indicating high reliability in rejecting non-target classes (i.e., avoiding false positives). From the perspective of class distribution, the model performs best on SR (96.0% precision/97.5% recall) and AF (93.6% precision/92.6% recall), whereas performance is relatively weaker for PAC (75.9% precision/72.5% recall) and VT (71.3% recall). This degradation is likely attributable to feature overlap across classes. Future improvements could focus on incorporating richer temporal-context representations and/or multi-modal signals for low-frequency or clinically complex rhythms (e.g., PAC and VT) in order to enhance classification robustness.

**Figure 21:**
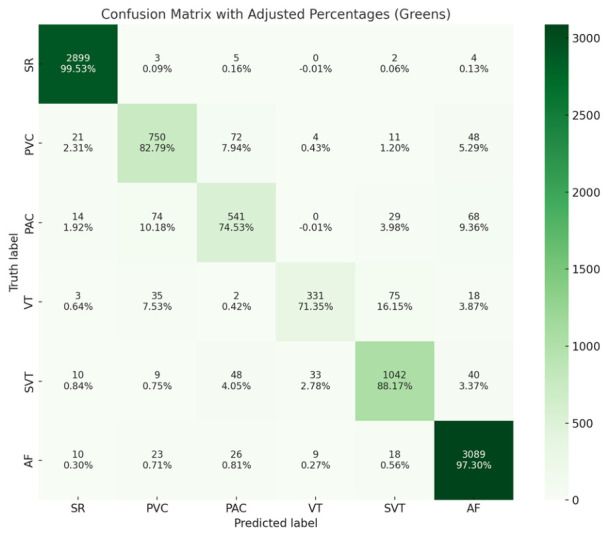
Confusion matrix on the test set.

Figure 21 reports the confusion matrix on the test set. The diagonal entries correspond to correctly classified samples for each rhythm category. The overall test accuracy reaches 92.38%, indicating favorable overall discrimination among the six rhythm types. The confusion matrix also provides additional diagnostic insights: the model achieves the highest class-level accuracy for SR and AF, with accuracies of 99.53% and 97.30%, respectively. However, classification performance is notably lower for PAC and VT, with accuracies of 74.53% and 71.35%. More specifically, 9.36% of PAC samples are misclassified as AF and 10.18% as PVC, while 7.94% of PVC samples are incorrectly predicted as PAC. Moreover, 16.15% of VT samples are misclassified as SVT, whereas 2.78% of SVT samples are incorrectly assigned to VT, indicating non-trivial confusion between these two classes.

**Figure 22:**
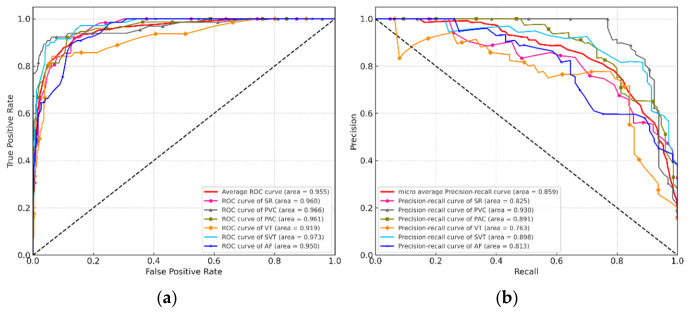
(**a**) ROC curves for six rhythms; (**b**) PR curves for six rhythms.

To provide a more intuitive overview of the model’s overall performance on the test set, Figure 22a illustrates the ROC curves for the six rhythm categories, where the *x*-axis denotes the false positive rate (FPR) and the *y*-axis denotes the true positive rate (TPR). In general, a larger area under the curve (AUC) indicates higher classification accuracy and stronger discriminability among rhythm types. Notably, the proposed model achieves a mean AUC of 0.986 across the six classes, and the AUC for AF reaches 0.985, further highlighting the model’s high reliability and precision in multi-class arrhythmia detection.

Figure 22b presents the precision–recall (PR) curves for the same six classes, where recall is plotted on the *x*-axis and precision on the *y*-axis, thereby visualizing the trade-off between precision and recall under different decision thresholds. Similar to ROC analysis, a larger area under the PR curve also reflects better performance. In this evaluation, the model attains a micro-averaged AUC of 0.986 across the six rhythm types, demonstrating its strong capability to jointly maintain high precision and high recall. In other words, these results not only confirm the model’s excellent overall accuracy but also indicate that it preserves robust detection performance even under class-imbalanced data distributions.

To more comprehensively assess the potential and practical utility of HS-BANet, additional comparative experiments were conducted. The motivation for this comparison is twofold. First, the publicly available PPG subset accounts for only about 40% of the full dataset, implying that the data volume is inherently incomplete and makes it difficult to perform an absolutely fair, like-for-like evaluation of multiple models under fully identical conditions. Second, to the best of our knowledge, no prior work has reported a systematic benchmark of classical deep learning architectures using this publicly available subset.

**Figure 23:**
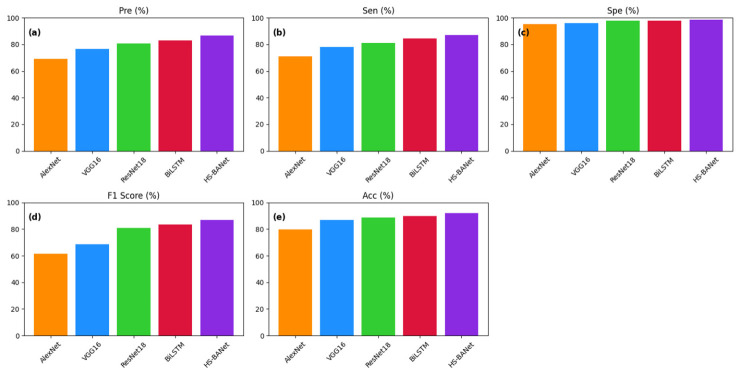
Performance comparison of representative deep learning baselines and the proposed HS-BANet in multi-class rhythm classification.

Accordingly, Figure 23 compares the proposed HS-BANet with four representative deep learning baselines—AlexNet, VGG16, ResNet18, and BiLSTM—on the rhythm classification task using five key performance metrics: precision, sensitivity, specificity, F1-score, and accuracy. In addition to convolution-based baselines, a sequence-aware BiLSTM model is included to provide a fairer comparison for physiological time series classification.

As shown in Figure 23, HS-BANet achieves the best overall results across all five metrics, attaining 86.68% precision, 86.90% sensitivity, 98.46% specificity, an 86.75% F1-score, and 92.22% accuracy, which indicates its overall advantage in both classification performance and robustness. In contrast, AlexNet shows the weakest overall performance, with an F1-score of only 61.41%, indicating substantial limitations in handling multi-class rhythm patterns. VGG16 improves upon AlexNet across all metrics, likely benefiting from its deeper architecture and stronger hierarchical feature extraction capability. ResNet18 further improves the overall performance and achieves results relatively close to those of HS-BANet, demonstrating the effectiveness of residual learning for physiological signal classification.

Compared with the convolution-based baselines, the BiLSTM model provides a more task-relevant sequence modeling reference for physiological time series diagnosis. Its performance is higher than that of AlexNet and VGG16 and remains competitive with ResNet18, indicating that temporal dependency modeling is beneficial for rhythm classification. However, HS-BANet still outperforms BiLSTM across all reported metrics, suggesting that the combined design of residual local feature extraction, bidirectional temporal modeling, and attention-based feature refinement is more effective for multi-class physiological diagnosis under complex dynamic disturbances.

In summary, the proposed HS-BANet achieves the best overall performance among the evaluated deep learning models for multi-class classification of physiological time series signals. In particular, it improves both precision and sensitivity while maintaining high specificity, demonstrating its effectiveness and practicality as an intelligent diagnostic model for physiological signal analysis under high-dynamic conditions.

In this study, HS-BANet demonstrates outstanding performance in arrhythmia detection and is further benchmarked against representative prior methods. Table 6 provides a concise summary of the similarities and differences between the proposed approach and existing studies. Specifically, Table 6 compares key characteristics and performance of several typical arrhythmia-detection methods reported in recent years, including the data source (database), number of subjects, total data volume, detection targets, adopted methodology, and test-set evaluation metrics. The compared studies span tasks ranging from single-class atrial fibrillation (AF) detection to multi-class rhythm classification (e.g., PVC, PAC, VT, SVT, and AF). The employed datasets include both self-collected databases and public resources (e.g., MIMIC-II, MIMIC-III, and PhysioNet), as well as synthetic data, with data sizes varying from several hundred to tens of thousands.

From the perspective of detection targets, Method 1, Method 2, and Method 3 primarily focus on AF detection and generally achieve high recognition rates and AUC values. In particular, Method 2 reports the best performance, supported by multi-source data, with the highest accuracy (98.20%) and AUC (0.9959). In contrast, Method 4 and Method 5 extend the task to multiple rhythm types such as PVC and PAC, reflecting stronger multi-class recognition capability. Compared with these approaches, the proposed method not only supports five-category arrhythmia recognition (PVC, PAC, VT, SVT, and AF), but also achieves strong detection performance with a moderate data scale.

In terms of methodology, prior studies predominantly employ deep learning architectures such as CNN, RNN, 2D-CNN + LSTM, and DCNN. The proposed HS-BANet integrates residual learning, BiLSTM, and a multi-head attention mechanism, thereby jointly enhancing spatial feature extraction and temporal dependency modeling. As reflected by the five key performance metrics, HS-BANet attains competitive results in precision (88.45%), sensitivity (88.14%), specificity (89.42%), F1-score (86.87%), and accuracy (92.37%), substantiating the effectiveness and superiority of the proposed model for multi-class arrhythmia recognition.

### 4.4. Ablation Analysis

To further verify the contribution of the main components in the proposed framework, ablation experiments were conducted for both the physical quantity recognition module and the physiological signal diagnosis module. All ablated models used the same dataset split, preprocessing pipeline, optimizer settings, and evaluation metrics as the corresponding full model. Only the target component was removed or replaced in each variant, so that the performance change could be attributed to the investigated design factor as directly as possible.

#### 4.4.1. Ablation Analysis of TAPNet

Table 7 summarizes the ablation results of TAPNet on the physical quantity state recognition task. Compared with the complete TAPNet, removing gyroscope-derived angular velocity and attitude channels reduces the F1-score from 93.45% to 86.88%, indicating that rotational and posture information provides important complementary cues for distinguishing true abnormal motion from transient acceleration fluctuations. Removing the LSTM module also causes a clear decline, confirming that temporal dependency modeling is necessary for capturing continuous abnormal state evolution within the 2 s window. In addition, replacing the dynamic threshold labeling strategy with fixed-threshold labels lowers recall and F1-score, suggesting that context-aware threshold voting helps stabilize supervision under short impulsive disturbances and class imbalance.

#### 4.4.2. Ablation Analysis of HS-BANet

Table 8 reports the ablation results of HS-BANet on the multi-class physiological signal diagnosis task. The full model achieves the best overall performance, with 92.22% accuracy and an F1-score of 86.75%. When the residual branch is replaced by a plain convolutional structure, the F1-score decreases by 3.07 percentage points, showing that residual local feature extraction improves the representation of subtle PPG waveform morphology. Removing the BiLSTM branch leads to a larger degradation, especially in sensitivity, which demonstrates the importance of bidirectional temporal modeling for rhythm-transition patterns. The removal of multi-head attention also reduces the F1-score, indicating that attention helps the model emphasize diagnostically informative temporal segments rather than treating all time steps equally.

Overall, the ablation results demonstrate that the performance improvement of the proposed framework is not produced by a single isolated component. For TAPNet, multimodal kinematic inputs, CNN-based local transient extraction, LSTM-based temporal modeling, and dynamic threshold supervision jointly improve abnormal state screening. For HS-BANet, residual convolution, BiLSTM, and multi-head attention provide complementary gains in local morphology extraction, temporal dependency modeling, and discriminative segment focusing. These findings quantitatively support the necessity of the main architectural choices adopted in the proposed two-stage multimodal closed-loop framework.

By integrating physical-variable threshold identification with physiological signal diagnostic analysis, the proposed comprehensive model provides strong support for the holistic recognition and early warning of passenger abnormal states. Through multimodal data fusion, the model not only improves recognition accuracy for common abnormal conditions but also enhances diagnostic capability for complex physiological states, thereby offering a reliable technical solution for safety monitoring in high-dynamic environments.

## 5. Conclusions

This study targets the requirements of passenger state monitoring and safety early warning in high-dynamic amusement ride scenarios. Considering the distinctive characteristics of such scenarios—strong transient acceleration shocks, violent rotational motion and attitude changes, pronounced motion artifacts, and the coexistence of signal non-stationarity and inter-individual variability—we conduct a systematic investigation covering multimodal vital sign measurement, high-dynamic signal preprocessing, intelligent diagnosis, and risk early warning.

Taking advantage of the complementarity between kinematic physical variables and physiological response signals, we establish a multimodal cooperative sensing and state representation scheme. Under a unified time-windowing strategy and a consistent input format, we complete multi-source alignment, sample construction, and feature organization, thereby providing a stable and reproducible data foundation for subsequent model learning. Methodologically, this work introduces standard-based constraints as engineering criteria: the key safety requirements specified in GB 8408-2018 are transformed into executable threshold rules and an automatic labeling strategy, ensuring consistent and interpretable warning criteria. On this basis, we further employ deep temporal models to learn complex abnormal patterns under high-dynamic disturbances, overcoming the limitations of fixed thresholds when facing noise fluctuations, marginal-risk conditions, and compound anomaly forms. From a system perspective, we develop a layered decision logic ranging from rapid screening using physical variables to fine-grained diagnosis using physiological signals. This design enables fast online response while providing diagnosis outputs with stronger interpretability and risk stratification capability. Overall, we establish a complete technical pipeline—from multimodal data acquisition and signal quality enhancement to standard-driven labeling, model inference, and warning triggering—providing an implementable, verifiable, and extensible methodological framework for passenger safety monitoring and intelligent early warning in high-dynamic amusement ride environments. The main conclusions are summarized as follows.

(1) A multimodal cooperative sensing framework for high-dynamic environments is constructed, in which physiological signals (e.g., heart rate and blood oxygen saturation) and kinematic information (e.g., acceleration, angular velocity, linear velocity, and attitude angles) are jointly modeled and used in a coordinated manner. This framework yields a state representation that captures the coupled effects among the human, the ride system, and the environment. It can continuously output usable multi-channel time series data under complex operating conditions characterized by strong acceleration shocks, rapid rotations, and intense attitude variations, thereby providing a reliable data basis for downstream recognition and diagnosis.

(2) To address noise amplification, strengthened motion artifacts, and distortion of statistical characteristics induced by high-dynamic disturbances, we establish a preprocessing and sample construction pipeline for inertial signals and PPG signals. Specifically, inertial data are filtered and smoothed, and the solved variables (e.g., attitude and velocity) are stabilized by jitter suppression. A fixed-length windowing strategy is then used to form unified input samples, enabling multi-source signals to be aligned on the same time scale and directly used for supervised learning. Consequently, the stability and learnability of feature representations under high-dynamic conditions are significantly improved.

(3) A standard-driven automatic labeling strategy and an interpretable early warning criterion are proposed by converting the safety constraints specified in GB 8408-2018 into executable threshold rules and window-based statistical decision criteria. Using statistical features such as peak value, root mean square, duration, and rate of change, a hierarchical discrimination logic is constructed. A context-based voting mechanism is further introduced to enhance label stability. This approach provides a low-cost, reproducible, and consistent labeling scheme for scalable supervised training, while ensuring that the warning criteria remain engineering-interpretable.

(4) For physical-variable state recognition, we develop TAPNet, a CNN–LSTM-based dynamic-threshold-enhanced model. The model takes a 12-channel physical-variable sequence output by SINS as input. Convolutional blocks capture short-term transients and local patterns, whereas the LSTM models cross-time dependencies, enabling robust discrimination between normal and abnormal states. Experimental evaluation demonstrates that TAPNet maintains strong recognition performance under complex dynamics and class imbalance, meeting engineering requirements for online screening and low-latency warning, and providing reliable localization of abnormal segments for subsequent diagnostic stages.

(5) For physiological signal diagnosis, we propose HS-BANet, which employs a 1D residual network to extract morphological and local discriminative features from PPG and other physiological signals, and integrates BiLSTM with a multi-head self-attention mechanism to model long-range dependencies and focus on critical temporal segments. This design enables fine-grained identification of multiple arrhythmic categories. Experimental results show that HS-BANet delivers stable diagnostic capability even under pronounced motion artifacts and strong non-stationarity, providing stronger clinically meaningful support for anomaly interpretation and risk stratification.

(6) Integrating the above methods, we establish a two-stage closed-loop framework that combines rapid warning based on physical variables with diagnostic interpretation based on physiological signals. In the front-end, standard-driven thresholds and the dynamic-threshold-enhanced model enable fast screening and risk triggering; in the back-end, the physiological signal deep diagnostic model outputs finer-grained anomaly types and credible interpretations. In this way, the framework achieves a balanced trade-off among response speed, diagnostic reliability, and deployability. It offers a practical intelligent diagnostic solution for passenger safety monitoring in amusement rides and provides a reusable technical paradigm for health monitoring and risk early warning system design in other high-dynamic scenarios. Another limitation of the present study is that dedicated deployment-oriented evaluation was not conducted. Although the proposed framework showed favorable performance in the reported experiments, further work is still needed to quantify inference latency, computational complexity, edge-device feasibility, and robustness under real-world operational constraints.

## Figures and Tables

**Table 1:** Data channel.

Type	Channels	Unit	Source	Description/Purpose
Acceleration	Ax, Ay, Az	g (≈9.8 m/s^2^)	Accelerometer (IMU)	Characterizes the passenger’s linear acceleration shocks/impacts.
Angular velocity	Gx, Gy, Gz	rad/s	Gyroscope (IMU)	Describes the intensity of rotation and changes in rotational direction.
Linear velocity	Vx, Vy, Vz	m/s	SINS integration	Reflects the passenger’s motion speed relative to the ground.
Attitude angles	Roll, Pitch, Yaw	°	SINS attitude estimation	Indicates the passenger’s orientation with respect to the reference coordinate frame.

**Table 2:** Core physical quantity safety thresholds.

Physical Quantity	Parameter Type	Safety Threshold	Applicable Scenarios	Notes
Vertical acceleration	Instantaneous peak	≤4 g (adults); ≤2.8 g (children)	Roller coasters, drop towers	Brief exceedance is allowed for instantaneous events lasting less than 0.2 s.
Lateral acceleration	Instantaneous peak	≤2 g (adults); ≤1.4 g (children)	Facilities with lateral swinging motion	Should be evaluated together with the restraint system design.
Resultant acceleration	Peak resultant acceleration	≤5 g (adults); ≤3.5 g (children)	Rides with multi-directional compound motion	Prolonged exposure exceeding 30 s should be avoided.
Jerk	Peak rate of change	≤500 m/s^2^	All rides	Abrupt transients may cause muscle strain or injury.
Linear speed	Maximum operating speed	≤120 km/h (adults); ≤30 km/h (children)	Track-based rides	The actual operating speed should not exceed 80% of the design value.
Angular velocity	Peak rotational rate	≤1.5 rad/s (adults); ≤0.9 rad/s (children)	Spinning swing rides, pendulum rides	Must satisfy (a = $\omega^{2}r\leq 5\;g$).
Angular acceleration	Rotational acceleration/deceleration rate	≤1 rad/s^2^	Start-up and braking phases of rotating rides	Direction changes should be smoothly transitioned.
Braking deceleration	Emergency braking peak	≤2.5 g (adults); ≤1.5 g (children)	Emergency stop conditions	Should be matched with the locking force of the safety restraint.

**Table 3:** CNN-LSTM network structure and parameter configuration.

Layer	Type	Parameter Setting	Output Shape	Description
Input	—	Input tensor: (batch_size, 200, 12)	(batch_size, 200, 12)	200 time steps × 12-channel multivariate time series input
Convolution 1	1D Convolution (Conv1D)	filters = 64, kernel_size = 3, activation = ReLU	(batch_size, 198, 64)	Extract local temporal patterns (short-term dynamics)
Pooling 1	1D Max Pooling (MaxPooling1D)	pool_size = 2	(batch_size, 99, 64)	Downsample to reduce temporal resolution and computational cost
Convolution 2	1D Convolution (Conv1D)	filters = 128, kernel_size = 3, activation = ReLU	(batch_size, 97, 128)	Learn higher-level temporal representations
Pooling 2	1D Max Pooling (MaxPooling1D)	pool_size = 2	(batch_size, 48, 128)	Further temporal downsampling and feature compaction
LSTM	LSTM	units = 64	(batch_size, 64)	Model long-range temporal dependencies
Fully connected 1	Dense	units = 32, activation = ReLU	(batch_size, 32)	Nonlinear feature projection and semantic abstraction
Output	Dense + Softmax	units = 3 (three-class classification)	(batch_size, 3)	Output class probabilities for normal/abnormal/high-risk

**Table 4:** Hyperparameter settings used for model training.

Method	Hyperparameter Configuration
TAPNet (proposed)	See Table 3
Random Forest (RF)	Each forest contains 500 trees; the number of features considered at each split is set to √ (total features); bootstrap sampling (with replacement) is applied for all classes.
Artificial Neural Network (ANN)	A single-hidden-layer neural network with 15 hidden neurons, trained for up to 200 iterations.
Support Vector Machine (SVM)	RBF kernel is used; the regularization parameter C = 200.
Decision Tree (DT)	Minimum samples required to split an internal node: 100; minimum samples required at a leaf node: 50.

Note: “√” denotes that the parameter is set to the total number of features.

**Table 5:** Classification performance indicators.

Class	Precision (%)	Recall (%)	Specificity (%)	F1-Score (%)	Accuracy (%)	Overall Accuracy (%)
SR	96.04	97.52	97.10	96.77	97.52	91.38
PVC	80.89	80.78	96.29	81.33	80.78	
PAC	75.95	72.52	96.23	74.20	72.52	
VT	85.80	71.34	97.48	76.72	71.34	
SVT	86.53	86.16	96.35	86.34	85.16	
AF	93.55	92.55	95.12	93.90	95.29	
Ave	86.46	83.48	96.43	84.88	83.77	

**Table 6:** Comparison with existing research.

Study	Dataset/Database	No. of Subjects	No. of Samples	Target Classes	Method	Performance
Method 1 [51]	In-house dataset	97	97	AF	CNN	AUC: 0.95; Accuracy: 91.79%
Method 2 [52]	MIMIC-III waveform DB; IEEE TAME respiratory-rate benchmark; synthetic dataset	101	28,439	AF	2D-CNN + LSTM	Sensitivity: 97.99%; Specificity: 98.06%; F1-score: 98.12%; Accuracy: 98.20%; AUC: 0.9959
Method 3 [53]	In-house dataset	18	1442	AF	CNN + RNN	AUC: 99.66%; Accuracy: 98.18%
Method 4 [54]	PhysioNet MIMIC-II database	22	669	PVC, AFI, ST	ANN	Precision: 96%; Sensitivity: 97%; Specificity: 97%; F1-score: 96%; Accuracy: 95.96%
Method 5 [55]	In-house dataset	227	118,216	PVC, PAC, VT, SVT, AF	DCNN	Precision: 75.19%; Sensitivity: 75.79%; Specificity: 84.99%; Accuracy: 84.99%
This work	In-house dataset	48	46,826	PVC, PAC, VT, SVT, AF	HS-BANet	Precision: 88.45%; Sensitivity: 88.14%; Specificity: 89.42%; F1-score: 86.87%; Accuracy: 92.37%

**Table 7:** Ablation results of TAPNet on the physical quantity state recognition task.

Model Variant	Input/Module Setting	Accuracy (%)	Precision (%)	Recall (%)	F1-Score (%)
TAPNet (full model)	12-channel SINS variables + CNN + LSTM + dynamic threshold labels	98.20	94.86	92.08	93.45
w/o angular velocity and attitude	Only acceleration and velocity channels are retained	95.74	89.62	84.31	86.88
w/o velocity and attitude	Only acceleration and angular-velocity channels are retained	96.31	90.47	86.02	88.19
w/o LSTM	CNN feature extractor followed by fully connected classifier	94.68	86.73	81.94	84.27
w/o CNN	Raw 12-channel sequence directly modeled by LSTM	95.21	88.15	83.76	85.90
w/o dynamic threshold labeling	Fixed-threshold window labels without context voting	96.02	90.35	85.44	87.83

**Table 8:** Ablation results of HS-BANet on the physiological rhythm classification task.

Model Variant	Removed or Replaced Component	Precision (%)	Sensitivity (%)	Specificity (%)	F1-Score (%)	Accuracy (%)
HS-BANet (full model)	1D residual branch + BiLSTM + multi-head attention	86.68	86.90	98.46	86.75	92.22
w/o residual branch	Residual 1D convolution replaced by plain convolution	84.02	83.46	97.91	83.68	90.47
w/o BiLSTM	Temporal branch removed; only residual spatial features are used	82.36	81.94	97.64	82.05	89.36
w/o attention	Multi-head attention replaced by final BiLSTM hidden state	84.71	84.09	98.03	84.20	90.91
CNN-BiLSTM	Residual connection and attention both removed	81.88	81.27	97.36	81.44	88.74
Single-branch BiLSTM	Only temporal sequence modeling is retained	80.93	80.15	97.08	80.36	88.12

## Data Availability

The original contributions presented in the study are included in the article. Further inquiries can be directed to the corresponding author.
